# Theta oscillations tag episodic memories for sleep-dependent consolidation

**DOI:** 10.1371/journal.pbio.3003938

**Published:** 2026-08-27

**Authors:** Dan Denis, Zhiyi Chen, Manroop Kaur, Benjamin Clayden, Thomas Schreiner, Scott A. Cairney

**Affiliations:** 1 Department of Psychology, University of York, York, United Kingdom; 2 Department of Psychology and Neuroscience, Baylor University, Waco, Texas, United States of America; 3 Research Department of Early Life Imaging, Centre for the Developing Brain, School of Biomedical Engineering & Imaging Sciences, King’s College London, London, United Kingdom; 4 Department of Psychology, Ludwig-Maximilians-Universität München, München, Germany; University of Glasgow, UNITED KINGDOM OF GREAT BRITAIN AND NORTHERN IRELAND

## Abstract

How does the brain select which experiences to consolidate into long-term memory? Numerous neurobiological frameworks suggest that certain memories are “tagged” at learning for consolidation during later sleep. However, experimental evidence of such a tagging mechanism in the human brain is lacking. Employing multivariate classification of human electroencephalography data, we reliably decoded brain states for episodic memories that are tagged at learning for consolidation across sleep or wakefulness. The tagging of memories for consolidation across sleep (but not wakefulness) was linked to 3–8 Hz theta rhythms during learning. The magnitude of this tagging-related theta response predicted the coupling of slow oscillations to sleep spindles during post-learning sleep (an established neural correlate of sleep-dependent memory processing). In turn, slow oscillation-spindle coupling was associated with better memory performance at the post-sleep test. These findings provide new insights into the neural mechanisms through which our brains determine which information is retained for the future.

## Abstract 2

Why some experiences are retained while others fade remains a central question in memory research. This study shows that theta-band activity during learning marks memories for sleep-dependent consolidation and predicts sleep oscillation dynamics that support stronger subsequent memory retention.

## Introduction

Our memories are not an unedited replay of daily events, but rather a showreel of our most salient experiences. This adaptive filtering of memory is thought to be driven by a “tagging” process at learning, such that certain memories are prioritized for consolidation during offline periods [1,2]. Selective memory processing is considered essential to prevent system overload and ensure that memories are preserved in accordance with our cognitive and emotional goals [1].

Sleep has long been implicated in memory consolidation and may play a unique role in the selective retention of memories tagged at learning [3]. Indeed, there is evidence that salience cues at encoding prompt selective memory processing during sleep; information that is perceived as emotionally arousing [4–6], linked to financial reward [7,8] or considered relevant for future events [9–11] appears to be preferentially strengthened in the sleeping but not waking brain (though see [12]). Numerous theoretical frameworks therefore position sleep as a point of memory triage, with memories being consolidated on the basis of neurobiological tags established at the initial learning phase [1–3,13,14].

Findings from recent animal studies are consistent with the idea that memories are tagged at learning for overnight consolidation. Huelin Gorriz and colleagues (2023) found that sleep replay of hippocampal place cells increased with the frequency of maze traversals during prior wakefulness, but decreased when the experience was already familiar, suggesting that repetition and novelty influence which memories are tagged for sleep-dependent memory processing [15]. Relatedly, Yang and colleagues (2024) found that sleep sharp-wave ripples (SWRs) replayed maze trajectories that were reactivated during wake SWRs linked to reward, characterizing a mechanism for selective memory strengthening during sleep [16]. However, evidence for an analogous mechanism in humans has yet to be established and represents an important translational gap in our understanding of sleep’s role in memory.

If the human brain tags certain memories for sleep-dependent consolidation at learning, then it should be possible to differentiate brain activity patterns at learning for memories that are tagged for consolidation over later sleep, relative to memories that are retained across wakefulness. Multivariate classification analyses can meet this need by decoding brain states that are based on distinct patterns of neural activity [17]. In the current context, memories retained across sleep-filled delays should be distinguishable from memories retained across wake-filled delays, based only on the neural operations (i.e., tags generated) at learning. We tested this hypothesis in the present study by employing electroencephalography (EEG) and multivariate neural classifiers to differentiate encoding trials for memories that were subsequently retained after sleep or wakefulness.

Although multivariate analyses can provide evidence of mnemonic tagging, they do not address the specific oscillatory rhythms that underpin this process. Theta oscillations (~3–8 Hz) are a promising candidate brain rhythm that may set the scene at learning for selective memory processing during later sleep. In humans, larger overnight memory gains are predicted by higher levels of theta activity at learning [18], mirroring animal research linking theta activity at wakefulness to replay during sleep [15]. Complementing these findings, other work has shown that the presence of reward or arousal cues at learning modulates theta activity in a manner that is predictive of subsequent memory [19,20]. Here, we isolated frequency-specific EEG activity in the theta band at learning to test the hypothesis that theta oscillations uniquely support the tagging of memories for sleep-dependent consolidation.

If theta oscillations at learning function as an instructional cue for overnight memory processing, they should also influence the neural signatures of sleep-dependent memory consolidation. Memories are thought to be reactivated during sleep via tightly coupled interactions between global slow oscillations (SOs; ~1 Hz), thalamocortical sleep spindles (~12–15 Hz) and hippocampal SWRs [21–25]. Growing evidence indicates that these sleeping brain rhythms, particularly sleep spindles, promote the selective strengthening of future-relevant information [8,9,26], and are correlated with theta power at learning [18]. Building on this work, we examined whether the magnitude of tagging-related (i.e., theta) activity at learning predicts the emergence of SO-spindle coupling during later sleep, with increases in SO-spindle coupling predicting sleep-related memory gains.

Participants completed a two-visit, within-subjects experiment where they learned word-object pairings before a 2-h sleep opportunity (nap) or an equivalent period of wakefulness. Using multivariate classification of EEG data at learning, we found evidence of distinct brain states for memories that were subsequently consolidated over sleep and those that were retained over wake. Moreover, theta power at learning was increased for memories consolidated across sleep (but not wake), with the magnitude of this theta response predicting the emergence of SO-spindle coupling, which in turn predicted memory retention. Together, these results support the view that memories are selectively tagged for sleep-dependent consolidation and suggest that this process is curated by theta oscillations at learning.

## Results

**Fig 1:**
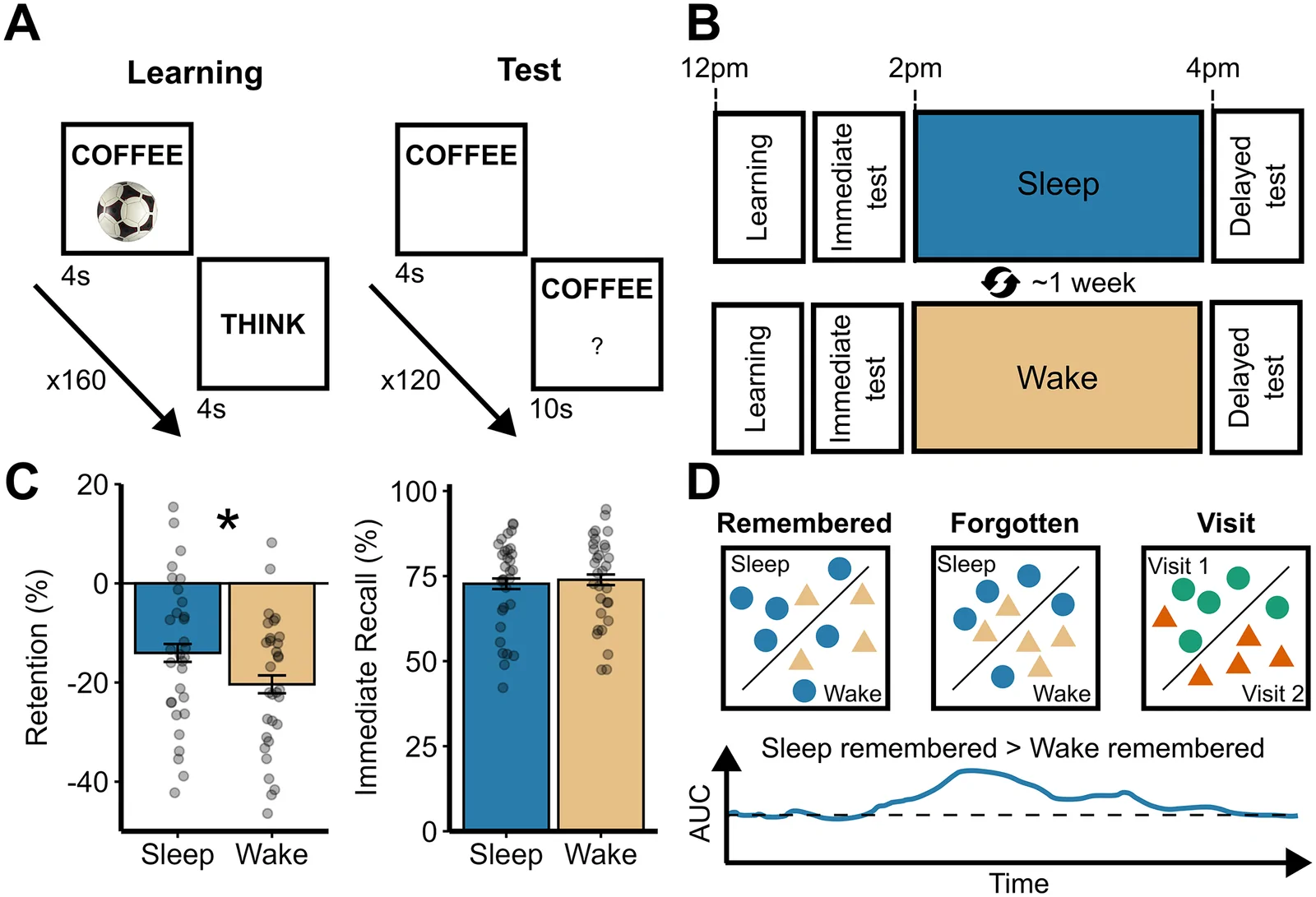
Experimental design. (A) During learning, participants were presented with 160-word object pairs. After viewing the word-image pair, participants were instructed to visualize an integrated scene combining the word-image items. During the test phase, participants saw 80 of the words presented at encoding, intermixed with 40 new words. For each word, participants first indicated whether the word was old or new. For each correctly identified old word, participants were asked to recall the associated image. A different set of old and new items was used at each test to avoid retrieval practice effects. (B) Memory was tested immediately (immediate recall) and again following a 2-h delay of either sleep or wake (delayed recall). Sleep and wake visits were manipulated within-subjects and performed one week apart in a counterbalanced order. (C) Retention (the change in associative memory recall at delayed compared to immediate recall, with a more negative number indicating more forgetting) was significantly better over the sleep delay compared to the wake delay (* = p < 0.05). There was no sleep/wake visit difference in the proportion of word-object pairs recalled at the immediate recall. Error bars indicate the standard error. (D) Multivariate classification approach. Multivariate classifiers were trained on the EEG time series at learning to distinguish trials that were later remembered after sleep from trials that were later remembered after wake. As control analyses, additional classifiers were trained to distinguish between trials that were forgotten in the sleep and wake conditions, and also to distinguish between experimental visits 1 and 2 (independent of the sleep or wake condition). For statistical analysis, a double subtraction was performed, meaning any classifier values significantly above zero reflected a memory-specific signature distinguishing between trials remembered after sleep and trials remembered after wake, independent of any non-specific session differences (AUC: area under the curve, the time series shown here is an illustrative example). The data underlying this figure are available at https://doi.org/10.17605/OSF.IO/TPWVB.

Thirty-one healthy young adults (*M*_age_ = 20 years, range = 18–23, 68% female; S1 Table) took part in the study. Participants began each visit by learning a set of word-object pairs (Fig 1A). Immediately after learning, and again following a 2-h delay (Fig 1B), participants were presented with the words in isolation, one after another, and for each word were instructed to make and old/new judgement and then recall the associated object image. To minimize any effects of retrieval practice on our behavioural measures of memory consolidation [27,28], participants were tested on different subsets of word-object pairs at the immediate and delayed tests. The 2-h delay was filled with either a daytime nap (S2 Table for sleep architecture) or time spent awake in the sleep laboratory watching nature documentaries (delay condition order counterbalanced).

Confirming the presence of a behavioural effect of sleep for memory, word-object (associative memory) retention was better when participants slept relative to when they remained awake (*t* (30) = 2.49, *p* = .019, *d* = 0.45; Fig 1C, left). Memory performance did not differ between immediate tests that took place before the sleep or wake delays (*t* (30) = −0.53, *p* = .60, *d* = −0.10; Fig 1C, right). There was no effect of visit number (visit 1 versus 2) on either immediate memory performance (t (30) = −2, *p* = .055, *d* = −0.36) or retention (*t* (30) = 0.16, *p* = .87, *d* = 0.03). There were no significant differences in old/new recognition scores between the sleep and wake conditions (S1 Text). Memory scores are shown in Table 1. Given the observed behavioural benefit of sleep for memory, we next turned our attention to tagging mechanisms at learning that pre-select memories for sleep-dependent consolidation.

**Table 1:** Memory scores.

	Immediate test		Delayed test		Retention	
	M	SD	M	SD	M	SD
Hit rate						
Sleep	90.92	5.96	85.81	7.09		
Wake	89.93	5.97	80.39	7.09		
False alarm rate						
Sleep	5.21	5.43	6.08	4.83		
Wake	5.15	5.52	6.12	4.83		
Associative memory						
Sleep	72.74	8.65	62.84	8.87	−12.33	8.96
Wake	73.91	8.65	59.15	8.88	−19.88	8.86

*Note*: M, mean; SD, standard deviation. Hit and false alarm rates refer to old/new judgements at retrieval. Associative memory was calculated as % of correctly identified old words for which the associated object image was also recalled. Retention was calculated within-subjects as [(delayed recall − immediate recall)/immediate recall].

### Brain activity patterns at learning differentiate memories that are retained over sleep or wake

If memories are selectively tagged at learning for consolidation during sleep, then it should be possible to distinguish between memories that are remembered after sleep or wakefulness based on unique patterns of brain activity at learning. To this end, we applied a multivariate linear classifier to the EEG data acquired at learning to distinguish between memories that were later recalled after sleep or wake. To isolate signatures of mnemonic tagging for subsequent sleep versus wake-based memory processing while accounting for non-specific between-session differences at learning, three separate classifiers were used. A *Remember* classifier was trained to distinguish between memories remembered after sleep and memories remembered after wake. A *Forgotten* classifier was trained to distinguish between memories forgotten after sleep and memories forgotten after wake. Finally, a *Visit* classifier was trained to distinguish learning at visit 1 from learning at visit 2 (independent of when the sleep or wake conditions occurred). Compared to surrogate decoders trained on randomized condition labels [25], all three classifiers achieved significant above-chance classification (all *p*_*cluster*_ < .003, see S1 Fig for classification time series).

We then isolated unique brain activity patterns at learning for memories that were subsequently recalled after sleep or wakefulness via a double-subtraction approach. First, the time series for the *Visit* classifier was subtracted from the time series for both the *Remembered* and *Forgotten* classifiers, generating visit-corrected signals of successful and unsuccessful learning, respectively. The corrected *Forgotten* classifier was then subtracted from the corrected *Remember* classifier, with the resultant signal distinguishing memories retained over sleep from memories retained over wakefulness (see the illustrative example in Fig 1D). This approach keeps the fate of the memory consistent between the sleep and wake conditions (i.e., all items were subsequently remembered) and is thus well suited for detecting mnemonic tagging for selective consolidation during later sleep.

**Fig 2:**
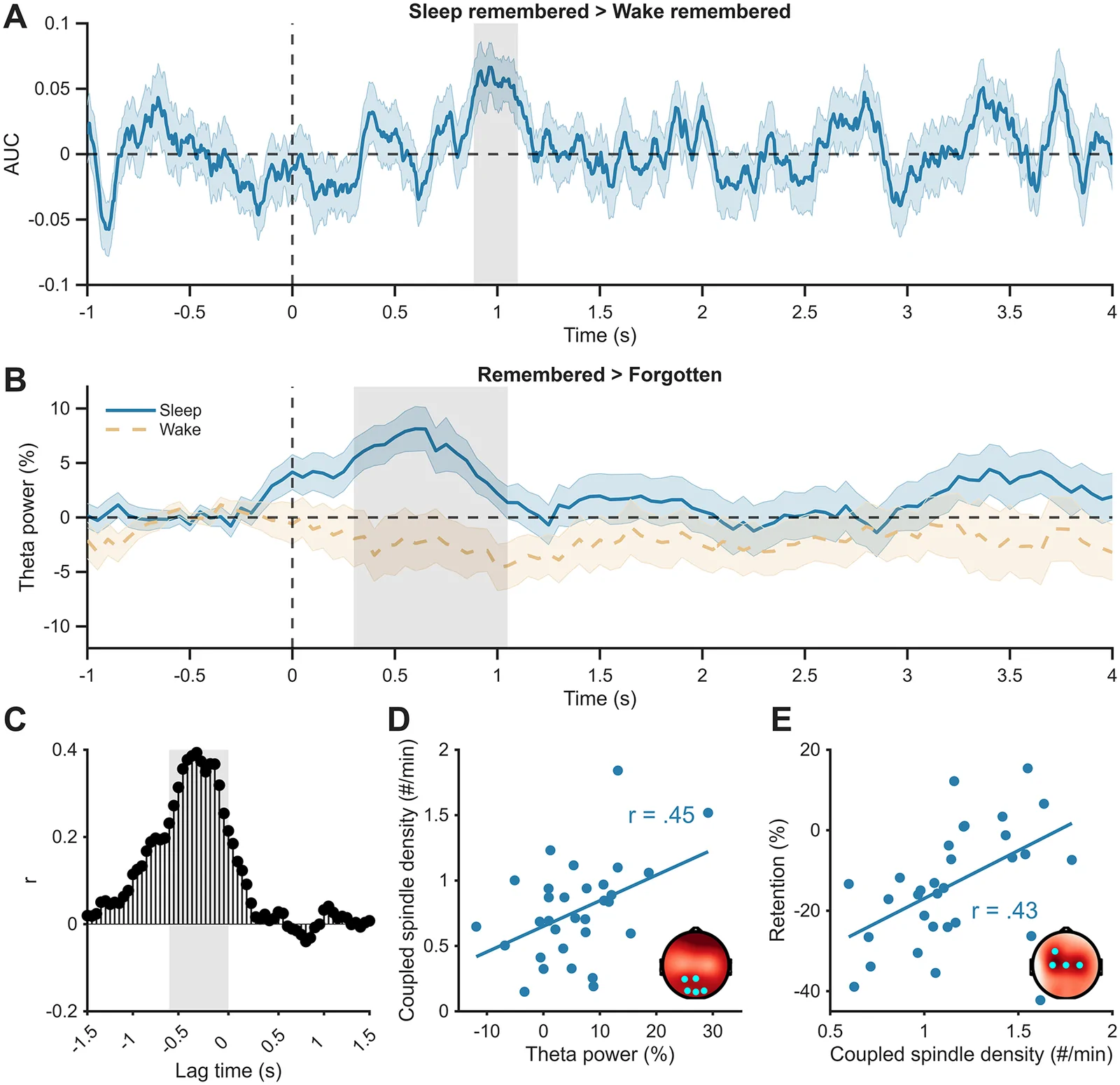
Neural activity at learning differentiates memories remembered after sleep or wakefulness. **(A)** Multivariate classification of EEG data at learning based on items that were subsequently remembered after sleep or wake. Significant classification above zero (extent of the cluster that is highlighted in gray, *p* < 0.05 corrected) indicates the time points of successful decoding. **(B)** Theta power at encoding (normalized within-subject and session as a % change relative to the pre-stimulus baseline) was significantly higher for items successfully recalled after sleep compared to wake (significant time points in the cluster are highlighted in gray, *p* < 0.05 corrected). **(C)** Cross-correlation between theta power during successful learning (sleep > wake) and the multivariate classifier time series. Significant correlations (highlighted in gray, false discovery rate adjusted) at negative lags indicate that increased theta power at learning in the sleep condition predicted successful multivariate classification 0.45s later. **(D)** Theta power at learning predicted SO-spindle coupling density during subsequent sleep. The topographical inset highlights significant electrodes in the cluster (*p* < 0.05 corrected). **(E)** SO-coupled spindle density predicted better memory retention after sleep. The topography inset highlights significant electrodes in the cluster (*p* < 0.05 corrected). The data underlying this figure are available at https://doi.org/10.17605/OSF.IO/TPWVB.

Significant classification was observed 0.87–1.13 s post-stimulus onset (*t*_sum_ = 125, *p*_cluster_ = 0.010, *d*_cluster_ = 0.72; Fig 2A). This shows that brain activity patterns linked to successful learning differed according to whether the memory was later remembered across sleep or wakefulness. Classifiers trained on trials that were subject to testing immediately after learning (i.e., *before* the sleep or wake delay) could not reliably differentiate between the sleep and wake conditions (*t*_sum_ = 82.30, *p*_cluster_ = 0.08; S2 Fig). Taken together, these findings are consistent with the idea that memories are tagged at learning for selective consolidation during later sleep. We next turned our attention to the brain rhythms supporting this tagging process.

### Theta oscillations support the tagging of memories for sleep-dependent consolidation

If theta oscillations underpin the tagging of memories for consolidation during later sleep, then theta activity during learning should support the processing of memories remembered after sleep, but not after wakefulness. To test this prediction, we decomposed the EEG data at learning into time–frequency representations (TFRs) and contrasted theta (3–8 Hz) power for subsequently remembered > forgotten trials, separately for the sleep and wake conditions.

Theta power at learning was significantly higher for word-object pairs remembered after sleep than word-object pairs remembered after wakefulness (0.3–1.05 s, *t*_*sum*_ = 42.53, *p*_*cluster*_ = 0.036, *d*_*cluster*_ = 0.54; Fig 2B). The increase in theta power was significantly greater than zero in the sleep condition (5.68% ± 8.09%; *t* (30) = 3.90, *p* < .001, *d* = 0.70), but not in the wake condition (−2.73% ± 12.18%; *t* (30) = −1.25, *p* = .22, *d* = −0.22). To confirm that our results were unique to the theta band, a broadband exploratory analysis was performed from 2 to 30 Hz, with no additional significant clusters emerging (S3 Fig for full TFRs and topographic effects, and S4 Fig showing no difference in event-related potentials nor in the tilt of the 1f aperiodic slope). No difference in learning-related theta power was observed between the sleep and wake conditions for trials successfully recalled at the immediate test (no clusters formed; S2 Fig). Taken together, these findings are consistent with the view that theta rhythms underpin the selective tagging of memories for consolidation during later sleep.

Interestingly, the peak in learning-related theta power for memories retained after sleep emerged ~0.4 s before the peak in multivariate classification accuracy for memories retained after sleep (theta power peak = 0.6 s; Fig 2B, classifier fidelity peak = 0.98 s; Fig 2A). This could imply that an early theta surge at learning triggers downstream processes that tag memories for consolidation during later sleep. To test for this, we adopted a cross-correlation approach which determined whether an early increase in theta power predicted later classification accuracy for memories retained after sleep. Consistent with this view, significant correlations were observed at lags ranging from −0.96 to −0.05 s, peaking at −0.45 s (*r*_max_ = 0.39; Fig 2C), aligning with the observed time difference in theta power and classifier peaks.

### Theta oscillations at learning predict slow oscillation-spindle coupling activity during sleep

If theta rhythms at learning represent a tagging mechanism for sleep-dependent consolidation, learning-related theta activity should be related to established oscillatory markers of sleep-dependent memory processing [2], namely the coupling between SOs and spindles (SO-spindle coupling [23]). We therefore predicted that theta power during successful learning would correlate with the density of SO-coupled spindles during later sleep, with the SO-spindle coupling density in turn correlating with memory retention after sleep.

SO-spindle coupling events were detected using automated detectors [21] (see S3 Table for spindle and SO metrics). Across all participants and electrodes, 16% ± 4% of spindles were coupled to an SO, far exceeding what would be expected by chance (S1 Text). We observed significant non-uniformity in the preferred phase of the SO to which spindles were coupled at 13/14 electrodes (*Z*s *>* 3.49, *p*s < 0.03). Sleep spindles preferentially coupled close to the positive peak of the SO (*M* = 20.96° ± 58.05° at electrode Cz; S5 Fig), in line with previous reports [21].

A robust linear regression model revealed a significant correlation between theta power during successful learning (i.e., for word-object pairs remembered after sleep) and SO-coupled spindle density (5 parieto-occipital electrodes, *t*_*sum*_ = 11.82, *p*_*cluster*_ = 0.028, *r*_*cluster*_ = .45; Fig 2D). In turn, memory retention across sleep was correlated with SO-coupled spindle density (4 fronto-central electrodes, *t*_*sum*_ = 10.62, *p*_*cluster*_ = 0.035, *r*_*cluster*_ = .43; Fig 2E). There was no direct relationship between theta power during successful learning and memory retention across sleep (*r* = −.03, *p* = 0.88). In a robust multiple regression model (Adjusted *R*^2^ = 0.19, *p* = 0.020), only SO-coupled spindle density emerged as a significant predictor of memory retention across sleep (B [95% CI] = 22.91 [6.05, 39.77], *p* = 0.010). Theta power was not an independent predictor of memory retention (B [95% CI] = 1.86 [−12.19, 15.91], *p* = 0.78).

To confirm the specificity of these results, we performed a series of sensitivity analyses. First, we found that theta power during successful learning did not predict the density of uncoupled spindles. Likewise, uncoupled spindles did not predict memory retention after sleep. Indeed, a multiple robust linear regression (Adjusted *R*^2^ = 0.21, *p* = 0.01) showed that SO-coupled spindle density uniquely predicted memory retention over sleep (B [95%CI] = 25.6 [8.63, 42.57], *p* = 0.005), independent of uncoupled spindle density (B [95% CI] = −0.75 [−4.16, 2.66], *p* = 0.65). Full cluster-based permutation results can be found in S4 Table.

As well as SO-coupled spindle density, previous work has found that the phase timing and consistency of SO-spindle coupling relates to memory consolidation [24]. However, we found no association between either coupling phase or consistency and overnight memory retention. A multiple robust linear regression (Adjusted *R*^2^ = 0.23, *p* = 0.02) showed that SO-coupled spindle density predicted memory retention over sleep (B [95%CI] = 23.40 [5.69,41.10], *p* = 0.012), independent of either mean coupling phase (B [95%CI] = 0.01 [−5.07,5.10], *p* = 0.99) or coupling consistency (B [95%CI] = 38.99 [−29.50,107.48], p = 0.25).

Sleep spindles (and their coupling to SOs) are known to be trait-like [29] leaving open the possibility that the observed correlations between theta power, SO-coupled spindle density and memory retention reflect more generalized individual differences than specific mnemonic tagging processes. However, theta power at learning for word-object pairs remembered after wakefulness was not significantly correlated with SO-spindle coupling density in the sleep condition. Similarly, theta power at learning for word-object pairs remembered at the immediate test in the sleep condition did not predict SO-spindle coupling density. Finally, no significant correlation was observed between SO-spindle coupling density in the sleep condition and retention in the wake condition (see S4 Table for full results). This suggests that our main reported effects correspond to a unique, sleep-specific tagging mechanism and not trait-like oscillatory interactions.

## Discussion

Memory tags established at learning are thought to direct consolidation processes during later sleep [1–3], but neurobiological evidence for such a tagging mechanism in humans has yet to be established. Our results address this gap in three ways. First, multivariate classifiers applied to EEG data acquired at learning reliably differentiated between memories that were remembered after sleep or wakefulness, consistent with a selective tagging process for sleep-dependent consolidation. Second, theta activity during successful learning was increased for memories remembered after sleep but not wake, highlighting theta oscillations as a candidate mechanism through which sleep-specific memory tags are generated at encoding. Third, the magnitude of theta activity at learning was correlated with the density of SO-coupled spindles during subsequent sleep, which in turn was correlated with memory retention. Taken together, these findings demonstrate that theta-modulated tags acquired at learning act as an instructional cue for selective, overnight memory processing.

Our results extend recent findings in the rodent literature to humans and outline a potential mechanism that triages experiences for sleep-dependent memory processing. While our results do not imply that consolidation processes are unique to sleep, they do suggest that there may be a unique memory function of sleep under certain conditions. Salience, novelty, and future relevance are all factors that influence overnight memory processing in animals and humans [6,15,16,30,31], and are thus likely involved in shaping which experiences get tagged for consolidation during sleep. We intentionally used non-affective stimuli in the current experiment so that we could isolate generalized neurocognitive markers of mnemonic tagging. However, individual differences in the perceived novelty or salience of word-object pairings may have influenced which memories were tagged for consolidation during sleep. Delineating the impact of such variables on our ability to decode neural tagging operations at learning is clearly a priority area for future research.

Theta oscillations have long been implicated in episodic learning and were uniquely associated with the tagging of memories for sleep-dependent consolidation in our current data. This builds on work in rodents showing that theta oscillations facilitate the late long-term potentiation necessary for selective memory strengthening [32,33], and research in humans demonstrating that theta activity at encoding maps onto the affective salience or distinctiveness of newly formed memories [19,20]. Mechanistically, theta oscillations are thought to support successful episodic learning by binding disparate aspects of an experience into a single, coherent representation [34,35]. Behavioural evidence has shown that the formation of integrated associations between previously unrelated items at encoding is key to observing a memory benefit of sleep [36–38]. Alongside this existing work, our findings might thus imply that theta-modulated, episodic binding is necessary for mnemonic tagging at learning and targeted processing during later sleep, with the propensity for such episodic binding influenced by the salience of the to-be-learned information.

Interestingly, evidence of tagging for sleep-dependent consolidation emerged in our classification analyses with a latency of ~1 s. This is temporally aligned with classifiers that have tracked the time course of memory reinstatement during retrieval in humans [39,40], and therefore suggests that neural tags for sleep-specific memory processing may emerge from early wake replay events. This notion is aligned with recent work in rodent models showing that awake replay shortly after a learning event itself constitutes a tagging mechanism for memory processing during later sleep [41]. In the context of the current study, early increases in theta activity and associated binding processes during episodic learning may prompt rapid replay events that constitute a tag for targeted overnight memory processing.

In further support of the idea that theta-generated tags act as an instructional cue for selective overnight memory consolidation, theta activity during successful learning was correlated with SO-spindle coupling density in parieto-occipital regions during subsequent sleep. This suggests that tags set at learning may influence SO-spindle coupling in learning-related areas [42]. In turn, SO-spindle coupling in fronto-central sites predicted the behavioural benefits of sleep for memory. Although speculative, it is possible that the topographical dissociation in SO-spindle coupling correlations reflects a gradual redistribution of memories from perceptual and learning-related (i.e., parieto-occipital) regions to longer-term (i.e., fronto-central) stores as sleep-dependent consolidation processes unfold [43,44]. This finding should be treated with caution, however, given the poor spatial resolution of scalp EEG and our experimental design not allowing for the assessment of the causal relationships between theta power at encoding, SO-spindle coupling, and memory consolidation

We focused on SO-spindle coupling during non-rapid eye movement sleep as a putative marker of sleep-dependent consolidation, based on long-standing evidence that these oscillations are involved in memory reactivation and systems-level consolidation [3,25]. However, there is evidence of mnemonic processing during rapid eye movement (REM) sleep, with some research suggesting that REM sleep supports the selective consolidation of emotionally salient memories [45–47]. A thorough examination of REM sleep in this dataset was not possible, owing to short REM sleep durations common to daytime napping paradigms. Follow-up research using an overnight design to sufficiently quantify REM sleep is therefore needed to understand how memory tagging operations interact with memory reprocessing during REM.

In the current study, sleep occurred soon after learning, making it unclear whether tags established at learning would endure over longer delays. Past research has found that sleep-memory effects are reduced over a 24-hour wake-first delay compared to a 24-hour sleep-first delay, which could be interpreted as learning-related tags fading over the initial wake interval [36,48–50]. These studies have typically used emotionally neutral stimuli such as word-pairs or object locations, meaning the initial tag would likely have been relatively weak. We hypothesize that a sufficiently strong tag at learning (e.g., corresponding to a highly salient or stressful experience) would elicit a strong enough tag for it to endure across a longer delay, permitting targeted consolidation when sleep is eventually reached.

Along similar lines, although our data suggest that memory tags established at learning steer consolidation during the first period of sleep after learning, it remains unclear how they affect memory operations across subsequent periods (i.e., nights) of sleep. Previous studies using targeted memory reactivation protocols (to bias sleep-dependent consolidation with learning-related sounds or odors) have shown that the effects of such interventions can take days or even weeks to emerge [51,52], meaning that tags formed at learning may likewise require multiple bouts of sleep to shape memory effectively. Furthermore, given recent views on the role of sleep in active forgetting [53], it is likewise possible that mnemonic tags established at learning may precipitate a targeted strengthening and *weakening* of prior experience to ensure that our memories remain aligned with our goals.

In sum, our findings provide evidence from multivariate neural classifiers that memories are tagged at learning for consolidation during later sleep. Mnemonic tagging is driven by theta oscillations, which act as instructional cues at encoding and thereby modulate sleep-dependent consolidation processes driven by the coupling of SOs and spindles. This serves as a mechanism through which we can retain our most formative experiences, allowing us to quickly retrieve goal-relevant memories in an adaptive and efficient manner.

## Materials and methods

### Participants

A total of 44 human participants enrolled in the study, of which 38 met inclusion criteria. Participants had no self-reported history of neurological, psychiatric, or sleep-related disorders, had normal or corrected-to-normal vision, were fluent in English, and performed adequately during a preliminary screening session (see Procedure: Preliminary session). Seven participants were excluded from the final analysis due to either not sleeping during their nap (*n* = 3) or being at floor or ceiling on at least one memory test (*n* = 4). Therefore, 31 participants were entered into the final analysis. Participants took part in exchange for an £80 e-voucher or course credit. The study was approved by the University of York Department of Psychology Research Ethics Committee and written informed consent was obtained from all participants. The study was conducted in accordance with the principles expressed in the Declaration of Helsinki. Data collection took place between 27th September 2022 and 16th March 2023.

### Stimuli

A set of 480 words and 320 images served as experimental stimuli. Words were all concrete nouns describing natural objects (e.g., plants, animals, foodstuffs). Images were all of manmade objects presented on a plain white background, and were taken from the Bank of Standardized Stimuli (BOSS) database [54].

### Procedure

#### Preliminary session.

Participants completed a preliminary visit to assess their eligibility for the study. After providing informed consent, participants completed questionnaires assessing their subjective sleep quality (Pittsburgh Sleep Quality Index [55]), morning-evening preference (Morningness–eveningness questionnaire [56]) and daytime sleepiness (Epworth Sleepiness Scale [57]). They then performed an initial memory assessment, where they learned associations between 84 semantically unrelated word pairs [36,37]. Each word pair was presented for 5 s, and participants were instructed to form a vivid mental image combining the two word-pair referents together in a scene. Immediately after learning, memory for all word pairs was tested via a cued recall procedure. The first word was presented on the screen, and participants had 10 s to respond with the second word in the pair. Participants scoring between 30% and 80% were invited back for the main experiment. This was done to minimize the risk of participants performing at either floor or ceiling in the main study [58].

#### Experimental sessions.

***Overview:*** The study consisted of two experimental sessions (sleep and wake condition) spaced 5–14 days parts. The order of the two conditions was counterbalanced across participants, and participants were informed that the current session involved a sleep or wake delay before the learning task began. Participants arrived at the sleep laboratory between 11am and 1 pm and completed a retrospective sleep diary for the previous three nights to assess sleep quality in the nights leading up to the experimental session. There were no differences in self-reported sleep quality in the nights prior to the sleep or wake visit (S1 Table). Next, participants were wired-up for EEG recordings. Following EEG setup, subjective alertness levels were assessed using the Stanford Sleepiness Scale (SSS) [59]. Participants were then familiarized with the images before learning the word-object pairs (see task details below) and performing an immediate test. Next participants either took a nap (2 h sleep opportunity; sleep condition), or remained awake in the lab for 2 h, watching nature documentaries (wake condition). Afterwards, subjective alertness was assessed again using the SSS, before memory performance was tested for a second time (delayed test). There were no differences in self-reported alertness levels at either assessment (S1 Table). All experimental tasks were presented using Psychtoolbox 3 (RRID:SCR_002881) [60] in MATLAB (RRID:SCR_001622, MathWorks, Natick, MA, USA). Across all experimental tasks, stimulus presentation order was randomized across participants.

***Familiarization:*** Participants were shown all of the to-be-encoded objects and their names/descriptors. This was done to facilitate subsequent word-object learning and to provide the proper object names for later cued recall. Each trial started with a fixation cross for 1 s (± 0.2 s jitter), followed by an object with its name presented above for 2 s. For each trial, participants were asked to think how often they would use or interact with the object in everyday life.

***Learning:*** Participants learned 160 word-object pairs. On each trial, a fixation cross was presented for 1 s (± 0.2 s jitter), followed by a word-object pair for 4 s. Participants were told to visualize an image that combined the referent of the word and the object into a single coherent scene (e.g., if the word COFFEE was paired with an image of a football, participants might visualize an image of a cup of coffee balanced on a football). This type of strategy has been shown to enhance sleep-dependent memory consolidation in previous work [36,37]. After 4 s, the word-object pair faded away from the screen and was replaced by an instruction to ‘Think’ for a further 4 s. During this period, participants were instructed to hold the mental image they had formed in their mind to facilitate successful learning [36]. At the end of the trial, participants were instructed to indicate the vividness of the mental image on a scale from 1 (no image formed) to 4 (very vivid visualization). Average visualization vividness was 2.78 ± 0.38 and did not differ between sleep and wake conditions (*p* = .58). Each trial was shown once during the learning phase, and participants were told beforehand that their memory for the associations would be tested.

***Test:*** One half of the word-object pairs were tested at the immediate test and the other half at the delayed test. This permits an assessment of sleep versus wake on memory retention while preventing retrieval practice effects that might emerge when testing the same items at the immediate and delayed assessments [28]. Other than that, the immediate and delayed tests were identical. Each test comprised 80 randomly chosen “old” words from the learning phase intermixed with 40 “new” words not seen by the participant during learning. Each trial began with a fixation cross (1 s ± 0.2 s jitter), after which a word was displayed on the screen for 4 s. During this time, participants were instructed to bring to mind the object originally paired with the word (if they recognized the word). After 4 s, participants indicated whether the word was “old” (i.e., seen during learning) or “new” (i.e., not seen during learning). A “don’t know” option was also presented. To minimize guessing, participants were instructed to only select “old” or “new” if they were confident in their judgement and to press the “don’t know” option when they were not confident. For each identified “old” trial, participants were then given 10 s to type a description of the object that was originally paired with the word.

***Sleep delay:*** Participants were given a 2 h opportunity to nap in a laboratory bedroom whilst EEG was monitored. After waking up, participants were given at least 10 min to mitigate the effects of sleep inertia before continuing with the post-sleep memory test.

***Wake delay:*** Participants remained in the sleep lab for 2 h watching nature documentaries. They were prohibited from other tasks such as reading or using their mobile phones.

***Electroencephalography:*** EEG was acquired with either an Embla NDx or Embla N7000 system using RemLogic 3.4 software. For all participants, the same EEG device was used for both experimental sessions. Gold-plated electrodes were attached to the scalp for EEG (14 electrodes, positioned according to the 10–20 system), above the right eye and below the left eye for EOG, and on the left and right side of the chin for EMG. Ground and reference electrodes were placed above the left and right eyebrow (corresponding to Fp1 and Fp2 of the 10–20 system). Finally, two electrodes were positioned on the left and right mastoid for offline re-referencing (see EEG analysis below). Data were recorded at 256 Hz (Embla NDx, later downsampled to 200 Hz) or 200 Hz (Embla N7000), and impedances were kept below 5 kΩ.

### Data analysis

#### Behaviour.

To assess memory performance, we calculated the proportion of correctly recalled objects relative to the number of correctly recognized words (i.e., the number of hits). In other words, object memory was only considered when participants correctly recognized the word. An object was considered correctly recalled if either 1) the participant typed the same descriptor that was shown during the familiarization phase or 2) the description provided unambiguously matched the object. Correctly recalling the object when cued with the word indicates successful associative memory retrieval, which was the focus here. For completeness, item memory scores (hit and false alarm rates) are provided in Table 1, and old/new recognition analyses are presented in the S1 Text. To quantify retention over the delay period, we calculated a relative change in object recall as [(delayed recall – immediate recall) / immediate recall]. This measure quantifies memory changes from the immediate to delayed test while controlling for immediate memory performance [36].

#### EEG.

***Preprocessing—wake:*** EEG data were preprocessed using functions from the FieldTrip (RRID:SCR_004849) toolbox for MATLAB (*ft_preprocessing*) [61] and custom MATLAB scripts. A notch filter was applied at 50 Hz to remove electrical line noise, and a high-pass filter was applied at .3 Hz to remove low- frequency artefacts. EEG channels were then re-referenced to the average of the two mastoid channels, before being epoched from −2 to +6 s around stimulus pair onset during learning. Noisy channels and epochs were identified and removed via visual inspection (*ft_visreject* function in FieldTrip; see S3 Table for trial counts) and bad channels were interpolated (range across participants: 0–3 channels, 90% of records had no channels interpolated) using a weighted average of the neighboring channels (*ft_channelrepair*). To correct for ocular artefacts, the epoched data were subjected to an independent component analysis, and ICA components reflecting eye blinks and movements were rejected.

***Preprocessing—sleep:*** Nap data were first manually scored in 30-s epochs in accordance with AASM criteria [62]. Artefactual epochs were detected using an automated algorithm [63]. For each EEG channel, we calculated per-epoch summary metrics of three Hjorth parameters (signal activity, mobility, and complexity). Any epochs in which any one of these parameters was >3 standard deviations from the mean, for one or more channels, were marked as artefact. Artefact detection was performed twice (in case of extreme outliers), and separately for each sleep stage (given inherent differences in the EEG signal between different sleep stages) [63]. Subsequently, all artefact-free non-rapid eye movement (NREM) sleep (stage N2 + N3) epochs were retained for further analysis.

***Multivariate analysis:*** We performed a linear classification of single-trial EEG data at learning using the MVPA-Light toolbox (RRID:SCR_022173) for MATLAB and an LDA classifier (using the *mv_classify_across_time* function) [64]. The classifier was trained to discriminate between learning trials for which the object memories were remembered in the sleep and wake conditions. Learning data were baseline corrected (−1 to 0 s relative to stimulus onset), linear trends were removed, and data were z-scored across all trials within each condition (sleep or wake) and for each time point separately. The two datasets were then smoothed using running average windows of 150 ms. The 14 EEG channels served as features and a different classifier was trained and tested on every sample from −1 to 4 s around stimulus onset. Area under the ROC curve was our classification metric, which indexes the mean accuracy with which a randomly chosen pair of learning trials could be assigned to their correct class (learning before sleep or wake; 0.5 = chance level performance). To avoid overfitting, data were split into training and test sets using a 5-fold cross-validation. Because cross-validation results are stochastic due to the random assignment of trials to folds, the analysis was repeated 10 times and the results averaged. To account for differences in the number of trials remembered after sleep compared to after wake, the number of trials per condition were equated by subsampling a random selection of trials from the higher trial count condition to match the condition with the fewer trials. This resulted in M = 32 (SD = 15; range = 19–52) trials per condition. To assess whether classifier accuracy was significantly above chance, surrogate decoding performance was calculated by shuffling the training labels 250 times, and taking the average of these permutations [25]. This provides, for each participant, a baseline decoding performance that can be compared to the classifier run on the real class labels. Multivariate classification was performed on the preprocessed EEG time series, rather than the theta time–frequency power time series (see next section), so as not to filter out potentially informative information at other frequencies present in the multivariate signal.

An important caveat of our approach is that, by necessity, the sleep and wake conditions were performed on separate experimental days. Non-specific between-session differences, such as differences in impedance levels and the exact positioning of electrodes could thus be picked up by the classifier. It was therefore crucial to ensure that any signal discriminating between the sleep and wake conditions did not more parsimoniously reflect significant classification of the experimental sessions. To account for this, a set of control analyses were carried out. First, we ran the classifier again, but this time distinguishing trials that were *forgotten* after sleep compared to *forgotten* after wake (i.e., the Forgotten classifier). By comparing this classifier to the first Remember classifier, we could look for periods where classification accuracy was significantly higher for later remembered versus forgotten trials. If classification accuracy was higher for one versus the other, this would speak against more generic between-visit differences, because we would not expect broad between-sessions differences to impact the memory conditions differently. As a second control analysis, we ran a third classifier to discriminate between visit 1 and visit 2 (the Visit classifier). This is dissociable from the sleep and wake condition because the assignment of visit order (sleep first or wake first) was counterbalanced across participants.

For our primary analysis, we performed a double subtraction of the classifier time series. First, we subtracted the Visit classifier time course from the Remember classifier and from the Forgotten classifier. Thus, any remaining values above zero would reflect genuine sleep versus wake condition differences for subsequently remembered and forgotten word-image pairings, having removed more generic between-visit factors. Second, we subtracted the visit-corrected Forgotten classifier time series from the visit-corrected Remember classifier time series. This resulted in a single time series where values above zero would reflect a remember-specific signal that differed depending on whether learning occurred prior to sleep or wake.

As a third control, we wanted to rule out any possibility that significant classification could be explained by participants simply engaging in a different cognitive strategy or being in a broadly different mental state (e.g., by knowing that they would be taking a nap or remaining awake across the delay). If this were the case, then significant classification between the sleep and wake conditions would also be possible on learning trials that were tested at the immediate, pre-delay test. Therefore, we repeated the above procedure but focused on learning trials that were remembered/forgotten at the immediate, pre-delay test. This control analysis was performed to confirm that effects were specific to testing after sleep.

***Time–frequency analysis:*** Preprocessed data were convolved with a five-cycle Hanning taper (*ft_freqanalysis* function in FieldTrip, using the *mtmconvol* method) from −2 to 6 s relative to stimulus onset in steps of 50ms, and from 3 to 30 Hz in steps of 1 Hz. To avoid edge artefacts, subsequent analyses focused on the −1 to 4 s time window (consistent with the classification analysis). Artefact rejection was performed on single-trial TFRs using a data-driven approach [58]: power values that exceeded the 85th percentile across all time/frequency points and trials were rejected and removed from subsequent analyses. TFRs were converted into a percent power change relative to a baseline interval of −0.4 s to −0.2 s before stimulus onset. This window was chosen to mitigate baseline contamination by post-stimulus activity while preserving proximity to stimulus onset [58]. Theta power estimates were extracted by averaging power values between 3 and 8 Hz. Then, the trial-averaged signal for subsequently forgotten word-object pairs was subtracted from the trial-averaged signal for subsequently remembered word-object pairs, separately for the sleep and wake delay conditions. Thus, the final TFR values entered into statistical analysis reflect theta power signatures of successful learning prior to a delay containing sleep or wake. To again confirm specificity to post-sleep testing, we repeated the above procedure but focusing on trials remembered/forgotten at the immediate, pre-delay test.

Spectral power estimates are made up of an oscillatory component and an aperiodic component following a 1/*f* power law. Recent evidence has suggested that changes in event-related theta power may reflect a tilt in the aperiodic slope, rather than differences in oscillatory activity [65]. To test for this, we estimated the aperiodic component of spectral power estimates during learning using irregular resampling auto-spectral analysis (IRASA [66]), implemented in FieldTrip using the *ft_freqanalysis* function with the method *irasa*, separately for the sleep and wake condition.

***Event-related potential analysis:*** To confirm that any differences between the sleep and wake condition could not be more simply explained by differences in the event-related potential (ERPs), ERPs for subsequently remembered and subsequently forgotten items following the sleep or wake delay were calculated using the *ft_timelockanalysis* function in FieldTrip. In line with previous research on subsequent memory effects, the preprocessed data underwent additional 15 Hz low-pass filtering [67]. Then, as with the TFRs, the trial-averaged signal for subsequently forgotten word-object pairs was subtracted from the trial-averaged signal for subsequently remembered word-object pairs, separately for the sleep and wake delay conditions.

***Slow oscillation-spindle event detection:*** All analyses were performed using the danalyzer toolbox for MATLAB (https://github.com/dandenis73/danalyzer) [21]. First, each individual’s peak spindle frequency was identified through visual inspection of the NREM power spectrum. The largest, most prominent peak in the 12–16 Hz range was considered that individual’s spindle peak frequency. Spindles were then automatically detected using a wavelet-based detector (*fun_sleep_spindles*) [21,68,69]. The raw EEG signal was convolved with a complex Morlet wavelet, with the wavelet peak frequency set at that individual’s spindle peak frequency, and the bandwidth of the wavelet (FWHM) set as a 1.3 Hz range centered on the peak frequency [21,70]. A spindle was detected whenever the wavelet-filtered signal exceeded a threshold of 6 times the median signal amplitude for a minimum of 0.4s. The threshold of 6 times the median has been empirically determined to maximize between-class (spindle, non-spindle) variance in the wavelet-filtered signal in previous work using a nap paradigm and healthy young adults [21].

Slow oscillations were then detected using a second automated algorithm (*fun_slow_oscillations*) [71]. Data were initially bandpass filtered between 0.5 and 4 Hz, and all positive-to-negative zero crossings were identified. Candidate slow oscillations were marked if two such consecutive crossings fell 0.8–2 s apart (i.e., 0.5–1.25 Hz, consistent with the SO frequency). Peak-to-peak amplitudes of all candidate oscillations were determined, and those in the top quartile (i.e., with the highest amplitudes) were retained as SOs. For SO-spindle coupling detection (*fun_so_spindle_coupling*), the Hilbert transform was applied to extract the instantaneous phase of the SO signal and the instantaneous amplitude of the spindle signal. For each detected spindle, its peak amplitude was determined. If the spindle peak was found to occur during the time course of any detected 0.5–1.25 Hz slow oscillation (i.e., between the two positive-to-negative crossings), the event was marked as a SO-spindle coupling event. Our primary measure of interest was the SO-spindle coupling density (i.e., the number of SO-coupled spindles per min of NREM sleep) [21,72,73]. For sensitivity analyses, we also extracted the mean coupling phase (degrees) and coupling consistency (measured as the mean length of the vector plotted in polar coordinates with a higher value indicating increased consistency in the SO phase to which spindles couple). Because coupling phase has a circular distribution, all phase values were converted to the absolute circular distance (using the *circ_dist* function of the circStats toolbox (RRID:SCR_016651) for MATLAB) from 0° [72,74]. This transformation rendered the variable linear.

### Statistical analysis

Differences between the sleep and wake conditions for (a) retention over the delay and (b) immediate recall performance were assessed with paired-samples *t* tests. For multivariate classification analyses, comparison of classifier accuracy against the surrogate baseline distribution was performed using a paired-samples *t* test at each time point. Analysis of the classifier time series following the double subtraction was achieved via a one-sample *t* test against zero at every time point. To examine sleep-wake differences in TFRs, between-condition differences were compared by way of a paired-samples *t* test conducted at every time point. Multiple comparisons across timepoints/electrodes, were controlled for using a cluster-based permutation method (either using the *ft_freqstatistics/ft_timelockstatistics* functions from *FieldTrip* or custom MATLAB code as appropriate) [75]. A cluster-corrected *p* < .05 was deemed statistically significant. Effect sizes were derived by averaging across all significant time/electrode points that contributed to the cluster, expressed as Cohen’s d.

To test for time-lagged associations between TFR power and multivariate classification, the cross-correlation between the TFR time series and lagged copies of the classifier time series (from −1.5 to 1.5 s lags, in 50 ms intervals) were computed using the *xcorr* function in MATLAB. Here, a negative lag would indicate that theta activity predicts later multivariate classification, whereas a positive lag would suggest the opposite. Multiple comparisons across multiple lags in the cross-correlation analysis were controlled using the False Discovery Rate [76]. Correlation analyses between theta power at learning, SO-coupled spindle density (number of coupled spindles per min), and memory retention were performed as robust linear regressions to minimize the influence of outliers using MATLAB’s fitlm function with RobustOpts turned on (implementing M-estimation for robust fitting). Multiple comparisons across electrodes were controlled using cluster-based permutation testing. Pearson’s r values (MATLAB *corr* function) are reported to quantify effect size.

## Supporting information

S1 Text Supplementary behavioural and SO-spindle coupling results. (DOCX)

S1 Table Participant demographics and questionnaire measures. (DOCX)

S2 Table Sleep architecture. (DOCX)

S3 Table Spindle and slow oscillations properties. (DOCX)

S4 Table Summary of cluster-based permutation testing results for associations between encoding theta power, SO-spindle coupling, and memory retention. (DOCX)

S5 Table Trial counts for EEG analyses. (DOCX)

S1 Fig Classifier performance versus baseline. Ability of classifier to decode learning trials into subsequently remembered across the sleep or wake delay (left); subsequently forgotten across the sleep or wake delay (middle); or decoding of experimental visit independent of delay condition or memory status (right). Classifier accuracy was compared to a surrogate decoding baseline, which was estimated by shuffling the training labels 250 times. Significant clusters (i.e., where classification exceeded chance levels) are highlighted in gray. Shaded areas around the lines indicate the standard error of the mean.
(TIF)

S2 Fig Multivariate classification and time-frequency representation for learning trials remembered at the immediate test: Top: Multivariate classification could not classify delay type (sleep or wake) based on learning trials that were remembered at the immediate test. Bottom: No significant difference in theta power at learning for trials recalled at the immediate test prior to the sleep or wake delay.
(TIF)

S3 Fig T **ime-frequency representations during learning for trials subsequently remembered after either the sleep (left) or wake (middle) delay.** Warmer colors indicate a greater % increase (relative to pre-stimulus baseline) in spectral power for remembered compared to forgotten trials. Contours indicate significant cluster (*p* < .05, corrected). The right-hand plot depicts the spatial extent of theta-related (3–8 Hz) learning activity for word-object pairs that were remembered (>forgotten) after sleep (>wake).
(TIF)

S4 Fig Event-related potentials and 1/f slope during learning*:* Left: Event-related potentials (ERPs) during learning. No significant differences in ERPs between the sleep and wake conditions. Shaded area around the line indicates the standard error. Right: 1/*f* aperiodic slope during learning (estimated using IRASA). No significant difference in the aperiodic slope between the sleep and wake conditions. Shaded area around the line indicates the standard error.
(TIF)

S5 Fig Temporal dynamics of SO-spindle coupling events. Top: The average time–frequency response of all SO-coupled spindles (−1.5 to 1.5 s, centered on the trough of the SO) with the time-domain averaged SO overlaid. Bottom: Histogram indicating the distribution of SO-coupled spindles, displayed as a percentage of all coupled spindles, binned into 100 ms intervals and averaged across all participants. All analyses were conducted at electrode Cz.
(TIF)

## Abbreviations

AUC: area under the curve
BOSS: Bank of Standardized Stimuli
EEG: electroencephalography
ERP: event-related potential
NREM: non-rapid eye movement
REM: rapid eye movement
SO: slow oscillation
SSS: Stanford Sleepiness Scale
SWRs: sharp-wave ripples
TFRs: time–frequency representations
