# Supplementary material for: Theta oscillations tag episodic memories for sleep-dependent consolidation
Source: PLoS Biol. 2026 Aug 27;24(8):e3003938. doi: 10.1371/journal.pbio.3003938 (PMC13521381; doi:10.1371/journal.pbio.3003938)
Supplement: Supplementary file 1 [file pbio.3003938.s001.docx]

**S1 Text**

## Recognition memory

We calculated d-prime (d’) as a general measure of recognition memory. The sleep and wake conditions did not differ on d’ scores at the immediate test (*t* (30) = 0.32, *p* = .75, *d* = 0.06). Similarly, there was no difference between conditions in the change in d’ across the delay interval (calculated as [(d’ delayed test – d’ immediate test) / d’ immediate test]; *t* (30) = -0.44, *p* = .66, *d* = -0.08).

## SO-spindle coupling

To determine whether the co-occurrence of slow oscillations (SOs) and spindles reflects a “true” coupling of the two oscillations, we needed to ensure that the number of coupling events exceeded what would be expected by chance, given the amount of time spent in non-rapid eye movement sleep, and the number of detected slow oscillations and spindles. To this end, the observed SO and spindle signal was compared to a randomized signal where the spindle signal was circularly shifted, and coupling to SOs was re-calculated [1, 2]. The start of the shifted spindle signal is set to a random position, and preceding entries are sequentially moved to after the end of the original signal. By shifting the spindle signal in this fashion, the temporal relationship between SOs and spindles is disrupted, whilst retaining the signal properties of the original signal (i.e., the distribution of SOs and spindles). This was performed over 1000 iterations to generate a null distribution of SO-spindle coupling. The null distribution was then compared with the observed number of coupling events. Across each participant/electrode, we calculated the percentage of participants and electrodes where the degree of observed coupling was significantly higher (p < .05) than what would be expected by chance. Across all electrodes, 29/31 (94%) of participants exhibited a rate of coupling that significantly exceeded what would be expected by chance (binomial test, p < .001).

1.	Denis D, Mylonas D, Poskanzer C, Bursal V, Payne JD, Stickgold R. Sleep Spindles Preferentially Consolidate Weakly Encoded Memories. J Neurosci. 2021;41: 4088–4099. doi:10.1523/JNEUROSCI.0818-20.2021

2.	Gilson M, Tauste Campo A, Chen X, Thiele A, Deco, G. Nonparametric test for connectivity detection in multivariate autoregressive networks and application to multiunit activity data. Netw Neurosci. 2017;1:357-380. doi: 10.1162/netn_a_00019
