# Supplementary material for: Theta oscillations tag episodic memories for sleep-dependent consolidation
Source: PLoS Biol. 2026 Aug 27;24(8):e3003938. doi: 10.1371/journal.pbio.3003938 (PMC13521381; doi:10.1371/journal.pbio.3003938)
Supplement: Supplementary file 2 [file pbio.3003938.s002.docx]

**S1 Table**

Participant demographics and questionnaire measures

|  | M | | SD | |  |  |
| --- | --- | --- | --- | --- | --- | --- |
| Age (years) | 20 | | 1 | |  |  |
| Sex | N | | % | |  |  |
| Female | 21 | | 68 | |  |  |
| Male | 10 | | 32 | |  |  |
|  | M | | SD | |  |  |
| PSQI | 4.45 | | 1.95 | |  |  |
| MEQ | 48.7 | | 7.38 | |  |  |
| ESS | 5.23 | | 2.88 | |  |  |
|  | Sleep visit | | Wake visit | | *t* | *p* |
|  | M | SD | M | SD |  |  |
| Sleep diary |  |  |  |  |  |  |
| TST (h) | 8 | 2 | 8 | 2 | 0.33 | .75 |
| SOL (min) | 20 | 22 | 21 | 29 | 1.28 | .21 |
| WASO (min) | 7 | 8 | 8 | 10 | 0.90 | .38 |
| SE (%) | 95 | 4 | 94 | 5 | 0.82 | .42 |
|  |  |  |  |  |  |  |
| Stanford sleepiness scale |  |  |  |  |  |  |
| Learning | 2.19 | 0.83 | 2.03 | 0.84 | 0.41 | .69 |
| Recall 2 | 2.81 | 1.01 | 2.71 | 1.19 | 0.33 | .75 |

*Note*. M = Mean, SD = standard deviation. PSQI = Pittsburgh sleep quality index global score. A higher score indicates worse subjective sleep quality (theoretical range: 0-21) MEQ = Morningness-eveningness questionnaire. A higher score indicates greater preference for mornings (theoretical range: 16-86) ESS = Epworth sleepiness scale. A higher score indicates higher subjective daytime sleepiness (theoretical range: 0-24). TST = total sleep time, SOL = sleep onset latency, WASO = Wake after sleep onset, SE = Sleep efficiency (% of time in bed that was spent asleep). Sleep diary measures calculated as the average of the three nights prior to the experimental day.
