# Supplementary material for: Theta oscillations tag episodic memories for sleep-dependent consolidation
Source: PLoS Biol. 2026 Aug 27;24(8):e3003938. doi: 10.1371/journal.pbio.3003938 (PMC13521381; doi:10.1371/journal.pbio.3003938)
Supplement: Supplementary file 3 [file pbio.3003938.s003.docx]

**S2 Table**

Sleep architecture

|  | M | SD |
| --- | --- | --- |
| Total sleep time (min) | 84 | 31 |
| N1 (%) | 13.52 | 8.25 |
| N2 (%) | 54.78 | 12.23 |
| N3 (%) | 19.98 | 14.59 |
| REM (%) | 11.71 | 12.07 |
| Sleep spindles (N) | 367 | 178 |
| Slow oscillations (N) | 303 | 134 |
| SO-spindle coupling events (N) | 57 | 28 |

*Note*. M = mean, SD = standard deviation. SO = slow oscillation. Spindle, SO, and SO-spindle coupling counts derived from electrode Cz.
