# Supplementary material for: Theta oscillations tag episodic memories for sleep-dependent consolidation
Source: PLoS Biol. 2026 Aug 27;24(8):e3003938. doi: 10.1371/journal.pbio.3003938 (PMC13521381; doi:10.1371/journal.pbio.3003938)
Supplement: Supplementary file 4 [file pbio.3003938.s004.docx]

**S3 Table**

Spindle and slow oscillations properties

| Metric | M | SD |
| --- | --- | --- |
| Spindle density (#/min) | 7.29 | 2.11 |
| Spindle amplitude (μV) | 33.37 | 8.21 |
| Spindle duration (s) | 0.86 | 0.07 |
| Spindle frequency (Hz) | 13.76 | 0.68 |
| SO density (#/min) | 5.94 | 0.84 |
| SO peak-to-peak amplitude | 109.17 | 35.58 |
| SO duration (s) | 1.05 | 0.04 |

*Note*. M = Mean, SD = standard deviation, SO = slow oscillation. All metrics derived from electrode Cz.
