# Supplementary material for: Theta oscillations tag episodic memories for sleep-dependent consolidation
Source: PLoS Biol. 2026 Aug 27;24(8):e3003938. doi: 10.1371/journal.pbio.3003938 (PMC13521381; doi:10.1371/journal.pbio.3003938)
Supplement: Supplementary file 5 [file pbio.3003938.s005.docx]

**S4 Table**

Summary of cluster-based permutation testing results for associations between encoding theta power, SO-spindle coupling, and memory retention

| IV | DV | # | t_sum_ | p_cluster_ |
| --- | --- | --- | --- | --- |
| Encoding theta (sleep condition) | Coupled spindle density | 5 | 11.82 | .028 |
| Encoding theta (sleep condition) | Uncoupled spindle density | - | - | - |
| Encoding theta (wake condition) | Coupled spindle density | - | - | - |
| Coupled spindle density | Memory retention (sleep condition) | 4 | 10.62 | .036 |
| Uncoupled spindle density | Memory retention (sleep condition) | - | - | - |
| Coupling phase | Memory retention (sleep condition) | 1 | -3.33 | .11 |
| Coupling consistency | Memory retention (sleep condition) | - | - | - |
| Coupled spindle density | Memory retention (wake condition) | - | - | - |
| Coupled spindle density | Memory retention (preliminary session) | - | - | - |

*Note*. # = the number of channels that formed a cluster, ‘-‘ indicates that no cluster was formed. All tests were robust linear regression models. *r* reflects the correlation magnitude averaged across all electrodes in the cluster.
