# Supplementary material for: Theta oscillations tag episodic memories for sleep-dependent consolidation
Source: PLoS Biol. 2026 Aug 27;24(8):e3003938. doi: 10.1371/journal.pbio.3003938 (PMC13521381; doi:10.1371/journal.pbio.3003938)
Supplement: Supplementary file 6 [file pbio.3003938.s006.docx]

**S5 Table**

Trial counts for EEG analyses

|  | Immediate test | | Delayed test | |
| --- | --- | --- | --- | --- |
|  | Remembered | Forgotten | Remembered | Forgotten |
|  | M (SD) | M (SD) | M (SD) | M (SD) |
| Sleep | 47 (16) | 38 (20) | 38 (18) | 36 (18) |
| Wake | 48 (15) | 38 (22) | 32 (15) | 41 (15) |

*Note*. M = mean, SD = standard deviation. Average trial counts presented for information only. Note that for all analyses, trial-counts were matched per-participant by randomly selecting a subset of trials from the higher count condition to match the lowest trial condition.
