# Supplementary material for: Theta oscillations tag episodic memories for sleep-dependent consolidation
Source: PLoS Biol. 2026 Aug 27;24(8):e3003938. doi: 10.1371/journal.pbio.3003938 (PMC13521381; doi:10.1371/journal.pbio.3003938)
Supplement: Supplementary file 7 [file pbio.3003938.s014.docx]

Reviewer 1

## Summary

This study aimed to identify neurophysiological signatures of memory "tagging" during learning that predict selective memory reprocessing during subsequent sleep. EEG-derived theta-band activity during encoding (3-8 Hz) differentiates items that will later be remembered after sleep versus wake. The magnitude of this theta response during learning predicts the density of slow-oscillation-coupled sleep spindles during the post-learning nap, a commonly used proxy for sleep-dependent plasticity, over parieto-occipital regions. SO-spindle coupling density over central regions is associated with better post-sleep memory performance.

## Overall evaluation

Overall, the manuscript is clearly written, focused, and communicates a strong central message. The experimental design is straightforward although some of the analytical decisions are in need of further justification. That said, the paper would benefit from additional integrative analyses to strengthen their interpretive claims (e.g., explicitly testing the proposed theta → coupling → behavior pathway). In addition, several important limitations of the paradigm and of inference from scalp EEG (especially regarding topographic differences and claims about tagging specificity) should be acknowledged more explicitly in the Discussion.

# Comment 1

In Figure 1D, the authors use three classifiers: remembered (sleep vs. wake), forgotten (sleep vs. wake), and visit (1 vs. 2). They are combined via a double-subtraction logic to isolate a tagging-related signal. While conservative, it is not obvious that this approach provides the most direct test of the tagging hypothesis as formulated in the Introduction.

If memories are "tagged" during encoding such that they are preferentially consolidated over others during sleep, in my opinion a more straightforward contrast remembered vs. forgotten items within each consolidation condition. Classifiers trained separately on remembered vs. forgotten trials for sleep and wake would allow a direct assessment of whether encoding-related signals are selectively enhanced for memories benefiting from sleep (i.e., sleep > wake). Under this logic, a separate visit classifier would not be strictly necessary if condition order was balanced across participants. The authors state in the respective methods section that their goal is to "distinguish memories retained over sleep from memories retained over wakefulness" (p. 7) which, in my opinion, is better expressed in a sleep > wake. This logic also applies to the theta power differences in panel Fig. 2B. Thus, please explain and justify your choice.

# Response

We thank the Reviewer for this comment. In the current study, we set out to test the theoretical perspective that memories are tagged during encoding for systems-level consolidation during later sleep (e.g., Stickgold & Walker, 2021; Cowan et al 2021). If this is the case, then memories that are tagged at encoding for sleep-dependent consolidation should show distinct neural signatures compared to memories that are retained across a wake-filled delay (where opportunities for systems-level consolidation are reduced). To provide evidence for this tagging hypothesis, we decided that the most appropriate analysis would be to directly contrast neural signals at encoding for memories that were remembered after sleep vs neural signals at encoding for memories that were remembered after wake (subtracting out neural signals at encoding for memories that were later forgotten after sleep/wake and condition order confounds (i.e., the ‘visit’ classifier). This means that the ultimate fate of the memory is consistent (remembered), and what is varying is what happens to these memories after encoding (i.e., they undergo systems consolidation during sleep or are presumably more passively protected during wake).

The Reviewer suggests an alternative approach to classification by contrasting remembered vs forgotten items within each consolidation condition (separately for sleep and wake) and then comparing whether the decoding signal is stronger for the sleep condition compared to the wake condition. This approach is akin to a subsequent memory effect (SME) which has previously been shown with decoders applied to EEG data (e.g., Noh et al., 2014). The analysis proposed by the Reviewer would therefore tell us if the magnitude of the SME varied between the sleep and wake conditions, but it doesn’t tell us whether there are distinct signatures at encoding for memories remembered after sleep compared to memories remembered after wake (and therefore does not test the hypothesis that certain memories are selectively tagged at learning for consolidation over sleep).

As noted by the Reviewer, we take a conservative approach by factoring out general effects of visit number, and also subtracting out information pertaining to forgotten items, ensuring our final analysis only includes memory-specific signatures of tagging. Given this, we have opted to keep our analysis approach unchanged from the original submission. However, given the Reviewer’s comment, we agree that a clearer justification of our classification approach is warranted. We have therefore added the following to the Results section where we outline our approach to classification:

*“We then isolated unique brain activity patterns at learning for memories that were subsequently recalled after sleep or wakefulness via a double-subtraction approach. First, the time series for the Visit classifier was subtracted from the time series for both the Remembered and Forgotten classifiers, generating visit-corrected signals of successful and unsuccessful learning, respectively. The corrected Forgotten classifier was then subtracted from the corrected Remember classifier, with the resultant signal distinguishing memories retained over sleep from memories retained over wakefulness (see the illustrative example in Figure 1D).* ***This approach keeps the fate of the memory consistent between the sleep and wake conditions (i.e., all items were subsequently remembered) and is thus well suited for detecting mnemonic tagging for selective consolidation during later sleep****”* – Page 6

# Comment 2

Related to the previous comment, to better understand the processes underlying state-specific ex ante tagging, it would also be important to know when participants were told whether they would be napping or not, and whether they were told (or expected) sleep/wake conditions to be counterbalanced.

# Response

Participants were not blinded to condition and knew that they would be sleeping in one of their visits and remaining awake the other. We have clarified this in the Methods as follows:

*“The order of the two conditions was counterbalanced across participants,* ***and participants were informed that the current session involved a sleep or wake delay before the learning task began****.”* – Page 13

This could plausibly lead to participants engaging in different cognitive strategies during learning before sleep or wake. However, if this were the case, then significant between-condition differences would emerge for items recalled both before and after the delay, as participants had no way of knowing which items would be assigned to the immediate and delayed tests. As we show in Figure S2, however, no sleep vs wake differences were seen for items recalled at the immediate test either in terms of multivariate classification or learning-related theta activity:

*“Classifiers trained on trials that were subject to testing immediately after learning (i.e., before the sleep or wake delay) could not reliably differentiate between the sleep and wake conditions (t_sum_ = 82.30, p_cluster_ = .08; Figure S2)”.* – Page 6

and

*“No difference in learning-related theta power was observed between the sleep and wake conditions for trials successfully recalled at the immediate test (no clusters formed; Figure S2).”* – Page 7

# Comment 3

A central claim of the manuscript is that theta-band activity during encoding serves as a memory tag that predicts subsequent sleep-dependent reprocessing, which in turn supports memory retention. However, this proposed pathway is currently supported only by two separate correlations: theta power with SO-spindle coupling (Fig. 2D) and SO-spindle coupling with retention (Fig. 2E).

Given the explicit directional logic of the model, a mediation analysis testing whether SO-spindle coupling mediates the relationship between encoding-related theta power and memory performance would substantially strengthen the conclusions. At minimum, it should be reported whether encoding-related theta power directly correlates with post-sleep retention, and how this relationship changes when spindle coupling is included as a predictor. If such analyses were not performed, the causal framing should be toned down and included as a limitation in the discussion.

# Response

We thank the Reviewer for this comment. We did consider a mediation analysis, but such analyses require relatively large sample sizes to be adequately powered (e.g. Fritz & MacKinnon, 2007). Given our sample size of N=31, we would likely be underpowered to detect a significant mediation effect. Nevertheless, we agree with the Reviewer that it would be useful to test whether encoding-related theta power directly contributes to post-sleep retention. We found no significant association. When both encoding-related theta power and SO-spindle coupling are included as predictors in a multiple regression model (with post-sleep retention as the outcome variable), only SO-spindle coupling emerges as a significant predictor. We have added these additional analyses to the results section as follows:

*“A robust linear regression model revealed a significant correlation between theta power during successful learning (i.e., for word-object pairs remembered after sleep) and SO-coupled spindle density (5 parieto-occipital electrodes, t_sum_ = 11.82, p_cluster_ = .028, r_cluster_ = .45;* ***Figure 2D****). In turn, memory retention across sleep was correlated with SO-coupled spindle density (4 fronto-central electrodes, t_sum_ = 10.62, p_cluster_ = .035, r_cluster_ = .43;* ***Figure 2E****).* ***There was no direct relationship between theta power during successful learning and memory retention across sleep (r = -.03, p = .88). In a robust multiple regression model (Adjusted R^2^ = .19, p = .020), only SO-coupled spindle density emerged as a significant predictor of memory retention across sleep (B [95% CI] = 22.91 [6.05, 39.77], p = .010). Theta power was not an independent predictor of memory retention (B [95% CI] = 1.86 [-12.19, 15.91], p = .78).****” – Page 8*

We have also noted in the discussion that our experimental design does not allow firm conclusions about causality to be drawn:

*“In further support of the idea that theta-generated tags act as an instructional cue for selective overnight memory consolidation, theta activity during successful learning was correlated with SO-spindle coupling density in parieto-occipital regions during subsequent sleep. This suggests that tags set at learning may influence SO-spindle coupling in learning-related areas [42]. In turn, SO-spindle coupling in fronto-central sites predicted the behavioural benefits of sleep for memory. Although speculative, it is possible that the topographical dissociation in SO-spindle coupling correlations reflects a gradual redistribution of memories from perceptual and learning related (i.e., parieto-occipital) regions to longer-term (i.e., fronto-central) stores as sleep-dependent consolidation processes unfold [43,44]. This finding should be treated with caution, however, given the poor spatial resolution of scalp EEG and our experimental design not allowing for the assessment of the relationships between theta power at encoding, SO-spindle coupling, and memory consolidation”* – Page 10-11

# Comment 4

To support the claim that theta-band activity reflects a sleep-specific tagging mechanism rather than general encoding strength, additional control analyses are warranted. Specifically, it would be informative to test whether encoding-related theta power also differentiates remembered vs. forgotten items at immediate retrieval, and whether this theta signal predicts SO-spindle coupling in the same way. Repeating analyses analogous to Fig. 2B and Fig. 2D using immediate memory outcomes would help establish that the observed theta-spindle relationship is selective to sleep-dependent consolidation rather than reflecting a general marker of successful encoding.

# Response

The analysis of encoding-related theta power for immediately recalled items can be found in the supplementary materials (no significant effects were found). We note this in the manuscript as follows:

*“No difference in learning-related theta power was observed between the sleep and wake conditions for trials successfully recalled at the immediate test (no clusters formed; Figure S2).”* – Page 7

We agree with the Reviewer that it is important to test whether encoding power related to recall on the immediate test predicts SO-spindle coupling. No association was found. These results are described in the manuscript as follows:

*“However, theta power at learning for word-object pairs remembered after wakefulness was not significantly correlated with SO-spindle coupling density in the sleep condition. Similarly, theta power at learning for word-object pairs remembered at the immediate test in the sleep condition did not predict SO-spindle coupling density..”* – Page 9

# Comment 5

Please specify the exact software and functions used for the statistical analyses, for instance for the (multivariate) robust linear regressions, to improve reproducibility.

# Response

We have gone through our methods section and made sure all software and functions used are clearly stated. For example, when describing the robust linear regressions we now write:

*“Correlation analyses between theta power at learning, SO-coupled spindle density (number of coupled spindles per min), and memory retention were performed as robust linear regressions to minimise the influence of outliers using* ***MATLAB’s fitlm function with RobustOpts turned on (implementing M-estimation for robust fitting).*** *Multiple comparisons across electrodes were controlled using cluster-based permutation testing. Pearson’s r values* ***(MATLAB corr function)*** *are reported to quantify effect size.”* – Pages 20-21

# Comment 6

Spindles occurring near the SO up-state (or down-to-up transition) are often considered most relevant for memory consolidation. Did the authors repeat the analyses using only up-state-locked spindles, and if so, were the effects comparable or stronger? If not, please justify the choice to include all spindles between SO zero crossings.

# Response

Thank you for this suggestion. To address the Reviewer’s question about whether up-state-locked spindles were more beneficial for memory consolidation, we ran an additional relationship examining the relationship between coupling phase and memory retention. This analysis can tell us whether spindles coupled to a particular SO phase are associated with superior memory retention. This analysis found no relationship between coupling phase and memory retention. This suggests that it is the occurrence of coupling, rather than the precise SO phase that coupling occurs, which is relevant to consolidation in this task. We have now added the coupling phase and consistency results to the manuscript. We now write:

*“As well as SO-coupled spindle density, previous work has found that the phase timing and consistency of SO-spindle coupling relates to memory consolidation [24]. However, we found no association between either coupling phase or consistency and overnight memory retention. A multiple robust linear regression (Adjusted R^2^ = .23, p = .02) showed that SO-coupled spindle density predicted memory retention over sleep (B [95%CI] = 23.40 [5.69 41.10], p = .012), independent of either mean coupling phase (B [95%CI] = 0.01 [-5.07 5.10], p = .99) or coupling consistency (B [95%CI] = 38.99 [-29.50 107.48], p = .25).”* – Pages 8-9

We also note that, at the group level, significant non-uniformity of preferred phase was observed, with spindles on average coupling to the “optimal” phase of the SO (near the peak). To visualise this more clearly, an illustration of SO-spindle coupling dynamics in the present sample have now been provided in Figure S5 of the Supplementary Materials as a peri-event time histogram. In our results we write:

*“We observed significant non-uniformity in the preferred phase of the SO to which spindles were coupled at 13/14 electrodes (Zs > 3.49, ps < .03). Sleep spindles preferentially coupled close to the positive peak of the SO (M = 20.96° ± 58.05° at electrode Cz;* ***Figure S5****), in line with previous reports [21].”* – Page 8

# Comment 7

Analysis of immediate vs. delayed performance (Fig. 2C): Rather than arguing for the absence of group differences at immediate recall, a more appropriate approach would be to include Session (immediate vs. delayed) and Condition (sleep vs. wake) in a single model (e.g., two-way ANOVA or linear mixed-effects model) and explicitly test their interaction. Please clarify whether the interaction would be significant in this case.

# Response

Running the analysis suggested by the Reviewer did not yield a significant interaction. It is important to note that this approach examines absolute performance at each memory test, which may be less sensitive to consolidation effects than the relative retention index we used in our analyses. By examining memory retention relative to immediate performance, we are better able to quantify how memories are consolidated across a delay (i.e., change between two time points), and this approach is widely used in the sleep and memory literature (e.g., Cordi et al., 2021; Denis et al., 2020; Schreiner et al., 2015; for just a few examples).

# Comment 8

The analysis for theta power was hypothesis driven. As a control, the authors also performed the classifications for the broadband spectra. Was the theta effect also present for this broadband analysis?

# Response

Yes, the theta effect remained significant in the broadband analysis. An outline of the significant cluster and the associated statistics have been added to Figure S3.

# Comment 9

For non-significant correlations involving other spindle, SO, or coupling parameters, described in the last paragraphs of the results section, please report p-values (and effect sizes where appropriate), either in the main text or supplementary materials, particularly when interpreting null findings.

# Response

Thank you for this suggestion. For ease of presentation, these have been provided in Table S4.

# Comment 10

Please provide a supplementary table summarizing spindle and SO characteristics (e.g., density, amplitude, frequency, duration) to support the validity of the detection procedures.

# Response

As requested, we have provided these data in Supplementary Table S3.

# Comment 11

The Discussion should explicitly address why SO-spindle coupling correlates with encoding-related theta over occipital regions but with behavioral retention over central regions. Given frequent claims that replay is local, this spatial dissociation requires cautious interpretation.

# Response

This is an interesting point that is worthy of further discussion. While speculative, one possibility is that these results reflect a shift in the neural basis of memory as sleep-associated consolidation processes unfold. We elaborate on this idea in the excerpt from our Discussion below:

*“In further support of the idea that theta-generated tags act as an instructional cue for selective overnight memory consolidation, theta activity during successful learning was correlated with SO-spindle coupling density in parieto-occipital regions during subsequent sleep. This suggests that tags set at learning may influence SO-spindle coupling in learning-related areas [42]. In turn, SO-spindle coupling in fronto-central sites predicted the behavioural benefits of sleep for memory. Although speculative, it is possible that the topographical dissociation in SO-spindle coupling correlations reflects a gradual redistribution of memories from perceptual and learning related (i.e., parieto-occipital) regions to longer-term (i.e., fronto-central) stores as sleep-dependent consolidation processes unfold [43,44]. This finding should be treated with caution, however, given the poor spatial resolution of scalp EEG and our experimental design not allowing for the assessment of the relationships between theta power at encoding, SO-spindle coupling, and memory consolidation.” – Pages 10-11*

# Comment 12

Replay processes also occur during wake. Please discuss how the apparent selectivity of encoding-related theta for sleep-dependent consolidation should be interpreted in this broader context.

# Response

This is an interesting point. Our results do not necessarily imply that replay is unique to sleep. Instead, our results suggest that there is a triage process whereby some memories are tagged for replay-mediated consolidation over sleep. This suggests that there may a unique function of sleep replay under certain conditions, which may be guided by cues such as salience, novelty, or future relevance. We speculate on these ideas in the discussion as follows:

*“Our results extend recent findings in the rodent literature to humans and outline a potential mechanism that triages experiences for sleep-dependent memory processing. While our results do not imply that consolidation processes are unique to sleep, they do suggest that there may be a unique memory function of sleep under certain conditions. Salience, novelty, and future relevance are all factors that influence overnight memory processing in animals and humans [6,15,16,30,31], and are thus likely involved in shaping which experiences get tagged for consolidation during sleep. We intentionally used non-affective stimuli in the current experiment so that we could isolate generalised neurocognitive markers of mnemonic tagging. However, individual differences in the perceived novelty or salience of word-object pairings may have influenced which memories were tagged for consolidation during sleep. Delineating the impact of such variables on our ability to decode neural tagging operations at learning is clearly a priority area for future research.”* – Pages 9-10

# Comment 13

The classifier distinguishes conditions only ~1 s after word onset and for a brief time window (~150 ms; Fig. 2A). Please discuss why tagging-related information emerges at this latency and why it is so temporally restricted.

# Response

We agree that additional discussion is warranted with regards to the time course of our classification result. A latency of 1s for significant classification is not unprecedented and is broadly in line with other work showing subsequently remembered paired associates can be discriminated from subsequently forgotten pairs with a ~1s latency (e.g., Kim et al., 2009). Given our cross-correlation analysis showed early increases of theta power predicted later classification accuracy, we speculate that our timing results could indicate that an early theta tagging signal (possibility responding to salience or novelty) triggers a downstream initial wake replay that captures the tag, prioritising the memory for sleep-dependent consolidation. This is in line with recent evidence from rodents suggesting immediate awake replay during a learning event constitutes a tagging mechanism for subsequent replay during sleep (van der Meer & Bendor, 2025). We now expand our discussion of these ideas in the manuscript as follows:

*“Interestingly, evidence of tagging for sleep-dependent consolidation emerged in our classification analyses with a latency of ~1 s. This is temporally aligned with classifiers that have tracked the time course of memory reinstatement during retrieval in humans [39,40], and therefore suggests that neural tags for sleep-specific memory processing may emerge from early wake replay events. This notion is aligned with recent work in rodent models showing that awake replay shortly after a learning event itself constitutes a tagging mechanism for memory processing during later sleep [41]. In the context of the current study, early increases in theta activity and associated binding processes during episodic learning may prompt rapid replay events that constitute a tag for targeted overnight memory processing.”* – Page 10

Regarding the finding that significant classification was so temporally restricted: this likely reflects the classifier capturing a transient stage at which the conditions are maximally separable, rather than implying that tagging itself is limited to a 150ms process.

# Comment 14

What was the range of trials per condition that could be used for analysis? (p. 17)

# Response

This has been added to the methods as follows:

*“To account for differences in the number of trials remembered after sleep compared to after wake, the number of trials per condition were equated by subsampling a random selection of trials from the higher trial count condition to match the condition with the fewer trials. This resulted in M = 32 (SD = 15; range = 19 – 52) trials per condition”* – Page 16

# Comment 15

The reasons provided for why the results do not reflect individual differences are not very convincing. It might be more interesting to test whether performance in the preliminary session was related to (coupled) spindle density.

# Response

Per the Reviewer’s suggestion, we correlated coupled spindle density with memory performance in the preliminary session test. We note, however, that this preliminary test is different to the main experiment (84 word pairs compared with 160 word image pairs). We found no significant association (no clusters formed), suggesting SO-spindle coupling during the post learning nap did not predict general memory performance at an earlier test. These results have been reported in Supplementary Table 4.

# Comment 16

The focus on correctly recalled, elaborate associations should be made more explicit in the title and the abstract.

# Response

We have made the following changes.

Title:

*“Theta oscillations tag episodic memories for sleep-dependent consolidation”*

Abstract:

*“Employing multivariate classification of human electroencephalography data, we reliably decode brain states for episodic memories that are tagged at learning for consolidation across sleep or wakefulness”*

We believe that these changes more accurately convey the nature of the memory task in abstract and title.

# Comment 17

What was the range of channels interpolated across participants? Given that only 14 electrodes were used, interpolation may have exaggerated the reliability of the signals.

# Response

The range of channels interpolated was 0 - 3 channels, with the vast majority of records (90%) having no bad channels interpolated. This has been reported in the manuscript as follows:

*“bad channels were interpolated (range across participants: 0-3 channels, 90% of records had no channels interpolated) using a weighted average of the neighbouring channels (ft_channelrepair). “* – Pages 15-16

# Comment 18

Did the theta effect also emerge from the broadband analysis that was used to explore correlations with other frequency bands?

# Response

We believe this is a duplication of Comment 8.

Reviewer 2

Thank you for the opportunity to review this manuscript on the role of scalp EEG theta activity in sleep-dependent memory consolidation. I read this work with great interest. The authors reported a single experiment with a cross-over, within-participants design, in which participants completed encoding and retrieval tests separated by a daytime nap or a period of wakefulness. Employing a variant of the subsequent memory effect approach and multivariate decoding (remember_sleep vs. wake decoding, forgotten_sleep vs.wake decoding, visit 1 vs. visit 2 decoding), results showed that theta power (but not other frequency bands) during encoding predicted memory performance in the sleep condition. Moreover, and perhaps most interestingly, theta power during encoding predicted subsequent slow oscillation - spindle coupling index during sleep, and then predicted sleep-dependent memory consolidation.

I have some comments about the rationale, analyses, and interpretation of the results.

# Comment 1

The role of theta power in episodic memories has received much attention in human memory research. It is almost well-established that theta power could predict various types of memory, at least during wakefulness (Herweg et al., 2020, Trends in Cognitive Sciences). This brings up the first question: What is the theoretical contribution of the current study? Although the study argues for the "memory tagging" mechanism, what the results showed were essentially the same as in previous research: enhanced theta power was associated with successful memory. Indeed, the study is probably the first demonstrating that theta activity predicted sleep-dependent memory consolidation, but then the results did not replicate the well-established findings that theta power could predict memory in wakefulness. Thus, the theoretical contributions remain unclear, and the significant discrepancy remains unresolved.

Conceptually, the manuscript argues that theta activity is a memory tagging mechanism. This is indeed an important question that still awaits more research. However, given that the behavioral and decoding analyses were performed at a condition level (remember vs. forgotten) instead of an item level, the results are not much different from previous findings showing that EEG/ERP activities during encoding were associated with subsequent retrieval (see work from Hanslmayr, Paller, Mecklinger on the EEG/ERP correlates of subsequent memory effects). The manuscript needs more elaboration on the novelty and how much new information the data provides.

# Response

We thank the Reviewer for their comments here. We agree that theta-related subsequent memory effects (SMEs) are indeed well established. However, the novelty of the present findings is not that theta power predicts memory per se, but that encoding-related theta power distinguishes memories retained after sleep vs wake while holding mnemonic outcome constant (remembered vs remembered; as opposed to remembered vs forgotten). This is conceptually different from a standard SME, and is instead addressing an entirely novel question of whether theta acts as a memory tag at encoding that selectively prioritises memories for consolidation across sleep (as opposed to across wakefulness).

To elaborate, researchers typically investigate subsequent memory effects (SMEs) by contrasting encoding trials that were subsequently remembered to trials that were subsequently forgotten. However, this is not the approach we took here. Instead, we directly contrasted memories remembered after sleep to memories remembered after wake. This means the ultimate fate of the memory is consistent (all items were subsequently remembered), and what is varying is what happens to these memories during the delay period (they either undergo systems consolidation during sleep, or presumably more passively protected during wake). As such, the novelty of this manuscript lies in our ability to detect unique patterns of activity at encoding coordinated by theta rhythms that specifically identify the memories that are to be selectively consolidated during sleep compared to wakefulness. There is, to our knowledge, no previous demonstration of this, and therefore our study provides the first evidence in humans that neural signatures during learning tag certain memories for consolidation during sleep, in-line with long-standing theoretical predictions that sleep selectively consolidates certain memories based on pre-sleep instructional cues.

We have added further clarification of why our design is well suited to address our research questions.

*“We then isolated unique brain activity patterns at learning for memories that were subsequently recalled after sleep or wakefulness via a double-subtraction approach. First, the time series for the Visit classifier was subtracted from the time series for both the Remembered and Forgotten classifiers, generating visit-corrected signals of successful and unsuccessful learning, respectively. The corrected Forgotten classifier was then subtracted from the corrected Remember classifier, with the resultant signal distinguishing memories retained over sleep from memories retained over wakefulness (see the illustrative example in Figure 1D).* ***This approach keeps the fate of the memory consistent between the sleep and wake conditions (i.e., all items were subsequently remembered) and is thus well suited for detecting mnemonic tagging for selective consolidation during later sleep*”** – Page 6

We have also changed the title of Figure 1D/2A to make it clearer that the significant contrast is Sleep remembered > Wake remembered

# Comment 2

The value and implication of the multivariate decoding approach need to be further elaborated. Given that the decoding analyses were all conducted at a condition level instead of an item/memory content level, how the decoding analyses can be informative beyond traditional condition/group-based EEG/ERP analyses remains unclear. Furthermore, given that there were no independent memory/functional localizer tasks (see Schreiner et al., 2021, Nature Communications) that can speak to memory content, how to best understand the decoding results and the theta activity remains unclear.

# Response

The Reviewer queries the added benefit of our MVPA approach over a traditional ERP approach. By taking a classification approach, we were able to take advantage of the multivariate nature of the EEG signature during each trial, which has greater sensitivity to distributed multichannel patterns even when no trial-averaged ERP is present. Such information would be lost with a traditional ERP approach, which typically involves heavy bandpass filtering (i.e., losing potentially important information) and averaging over trials. As we report in the manuscript, no difference between conditions was found for the trial-averaged ERP (Figure S4 of the manuscript).

The Reviewer also notes that no functional localiser task was used in the present study. Although functional localisers are needed for decoding memory content (e.g., objects vs scenes) during memory reinstatement, this was not the goal of this study. Instead of decoding content, we instead set out to decode the ultimate fate of a memory at a later timepoint filled with either sleep or wakefulness. Hence, the classifier is differentiating between brain states at learning for memories that are remembered after an interval of sleep or wake (our central research question, see our response to comment 1). A functional localiser is not warranted because it is unclear what such a localiser would be trained on.

# Comment 3

There are concerns regarding the precision of terms and quantification of the key neural activity, theta activities. The manuscript used theta power, theta oscillation almost interchangeably. However, recent methods can effectively quantify the theta oscillation from the background EEG spectral slope. To provide new theoretical insights, at least the analyses shall try to dissociate oscillatory activity from the background slope or the "tilt" (again, see Herweg et al., 2020, Trends in Cognitive Sciences), to better qualify the strong argument on the role of theta in so-called "memory tagging" for subsequent sleep-dependent memory consolidation.

# Response

The Reviewer suggests that our theta power effects may reflect a change in the tilt of the aperiodic spectral slope, rather than true oscillatory activity. We agree that this is important to address. Therefore, we estimated the aperiodic component of the power spectrum during learning trials separately for the sleep and the wake condition using irregular resampling auto-spectral analysis (IRASA). If the reported difference between sleep and wake conditions was due to a tilt in the 1/f slope, we should find a significant difference between conditions in the aperiodic component. However, no such difference was found (see Figure S4 of the revised manuscript), suggesting our effects reflect differences in oscillatory activity. We have added this analysis to methods section as follows:

*“Spectral power estimates are made up of an oscillatory component and an aperiodic component following a 1/f power law. Recent evidence has suggested that changes in event-related theta power may reflect a tilt in the aperiodic slope, rather than differences in oscillatory activity [65]. To test for this, we estimated the aperiodic component of spectral power estimates during learning using irregular resampling auto-spectral analysis (IRASA [66]), implemented in FieldTrip using the ft_freqanalysis function with the method irasa, separately for the sleep and wake condition.”* – Page 18

And in the results we write:

“*Theta power at learning was significantly higher for word-object pairs remembered after sleep than word-object pairs remembered after wakefulness (0.3 – 1.05s, t_sum_ = 42.53, p_cluster_ = .036, d_cluster_ = 0.54; Figure 2B). The increase in theta power was significantly greater than zero in the sleep condition (5.68% ± 8.09%; t (30) = 3.90, p < .001, d = 0.70), but not in the wake condition (-2.73% ± 12.18%; t (30) = -1.25, p = .22, d = -0.22). To confirm that our results were unique to the theta band, a broadband exploratory analysis was performed from 2-30Hz, with no additional significant clusters emerging (Figure S3 for full TFRs and topographic effects, and Figure S4 showing no difference in event-related potentials* ***nor in the tilt of the 1f aperiodic slope****).* – Page 7

# Comment 4

One novel result is that the theta activity during wakeful encoding was related to SO-spindle coupling during sleep. The current study calculated SO-spindle coupling density. However, a recent meta-analysis suggests that coupling percentage was a poor predictor of memory consolidation. Instead, the coupling strength and coupling phases are stronger and more reliable predictors (Ng et al., 2025, elife). The results and argument will be more convincing if theta activity can also predict coupling strength/phase, and then predict memory consolidation.

# Response

We would like to point out that our measure of SO-spindle coupling was not the coupling percentage (the % of spindles that couple to an SO) as used in the Ng et al., 2025 meta-analysis. Instead, we looked at the coupled spindle density (coupled spindles/per minute of sleep). Importantly, we were able to show in our results that coupled spindles uniquely predicted memory consolidation independently of uncoupled spindles:

*“Indeed, a multiple robust linear regression (Adjusted R^2^ = .21, p = .01) showed that SO-coupled spindle density uniquely predicted memory retention over sleep (B [95%CI] = 25.6 [8.63, 42.57], p = .005), independent of uncoupled spindle density (B [95% CI] = -0.75 [-4.16, 2.66], p = .65).”* – Page 8

This metric of coupling occurrence is conceptually distinct to the % coupled metric using in the Ng et al meta-analysis because we could directly compare the contribution of coupled and uncoupled spindles in a single model, and our results align with a multitude of other studies showing the unique importance of coupled spindle density as a predictor of memory consolidation independent of uncoupled density (e.g., Denis et al., 2021; 2025, Mylonas et al., 2020, Baxter et al., 2023; Solano et al., 2022). Given this prior literature, our choice of coupling metric was particularly informative for the present question. For completeness, we now report associations between coupling strength/phase and memory retention in the results as follows:

*“As well as SO-coupled spindle density, previous work has found that the phase timing and consistency of SO-spindle coupling relates to memory consolidation [24]. However, we found no association between either coupling phase or consistency and overnight memory retention. A multiple robust linear regression (Adjusted R^2^ = .23, p = .02) showed that SO-coupled spindle density predicted memory retention over sleep (B [95%CI] = 23.40 [5.69 41.10], p = .012), independent of either mean coupling phase (B [95%CI] = 0.01 [-5.07 5.10], p = .99) or coupling consistency (B [95%CI] = 38.99 [-29.50 107.48], p = .25).”* – Pages 8-9

Reviewer 3

Denis and colleagues examined whether learning paired associates imparted a specific tag that was related to sleep consolidation vs wake consolidation and how these tags might be associated with sleep oscillations known to be related to memory and subsequent retrieval. The study is well done and the paper is well written. Results show significant distinctions between remembered items across wake and sleep but not across immediate test, which is a critical control. I believe these findings will open new avenues of exploration between waking brain activity and subsequent sleep consolidation. I have some questions about the interpretation and some requests for further analyses.

# Comment 1

Although the results are quite clear, there is a little confusion in the writing about the results that can hopefully be cleared up. Such as this sentence: these findings are consistent with the idea that memories are tagged at learning for selective consolidation during later sleep. When I read that, it sounds like the brain is tagging material that it already knows will be consolidated by sleep and not wake. I assume this is not what the authors intend. Can you find a way to talk about this with less of a mindreading implication? For instance, the learning material is tagged at encoding irrespective of what consolidation process follows, and that the retrieval state of the memory tag is transformed by the circumstances of consolidation: sleep vs wake.

Now reading the discussion I see that you do in fact mean that the brain triages memories for sleep and not wake… hmm… the results show some benefits of sleep over wake for retention, but they are not massive benefits. And this was a simple pair associates task without a lot of complicated relations to remember. Therefore, the idea that some information should be stored away for a time when sleep can consolidate while most of the memories can be tagged and processed during either wake or sleep, seems like a stretch in logic to me. If a 24 hour protocol was used with a sleep first (trained at night) vs wake first (trained in am) comparison, would the authors predict that the theta-save-it-for-sleep effect would be eliminated because both groups eventually slept? Or would the authors say that the tag would decay?

# Response

Thank you for raising these important points. During the initial conception of this study, we did indeed set out to test the hypothesis that memories are tagged during learning for subsequent consolidation during sleep. This hypothesis has been part of active systems consolidation theory for some time (e.g., Born & Wilhelm, 2012), and has become central to many subsequent theories of sleep-dependent consolidation, especially theories concerning the selective nature of consolidation (e.g., Saletin & Walker, 2012; Stickgold & Walker, 2013; Kim et al., 2020; Cowan et al., 2021). We therefore set out to test for (as far as we know the first time) a neural mechanism that might support memory tagging for sleep-specific processing. We intentionally chose a simple paired-associates task where we could contrast recalled after sleep vs recalled after wake with all other things remaining equal, as we felt that would give us the purest estimate of a tagging signal (and not something that could be confounded with emotional valence/arousal, for example). However, the un-affective nature of the stimuli and relatively high trial count required for EEG analysis could have contributed to a relatively modest behavioural benefit of sleep (although still statistically significant).

As well as this theoretical basis, the logic of our reasoning is supported by recent research in rodents showing that hippocampal replay events during waking experience are predictive of those experiences being subsequently replayed during sleep (e.g., Huelin Gorriz et al (2023); Yang et al (2024)). It has recently been proposed that the initial wake replay sets a tag by modulating the latent excitability of the recently formed memory trace, and this excitable state then increases the likelihood that the experience will be replayed during later sleep (van der Meer & Bendor, 2025). Indeed, novelty and salience both increase the rate of awake replay, which would enhance the excitability and thus the strength of the tag. Relating this to our own data, we found evidence that the magnitude of the theta response during pre-sleep learning reflected the “strength” of the tag set. A larger theta response predicted increased SO-spindle coupling during sleep, an oscillatory event that clocks memory reactivation (Schreiner et al., 2021). Therefore, this suggests that as the theta tagging signal increased, SO-spindle coupling increased, providing more opportunities for memory reactivation to occur.

Although we did not directly manipulate salience/novelty of the stimuli in this study, it is likely that individual differences exist with regards to which individual trials participants found particularly salient. For example, a participant whose favourite football team won against their rivals the previous weekend may find an image of a football more emotionally salient than a participant who has no interest in football. As such, relatively more salient information may have led to a stronger theta response, tagging those memories for consolidation during sleep. Now that the present study has established foundational evidence supporting the idea of sleep-dependent memory tagging in humans, the results clearly pave the way for future research where salience is manipulated experimentally to alter neural tagging processes during learning. We present these ideas in the discussion as follows:

*“Our results extend recent findings in the rodent literature to humans and outline a potential mechanism that triages experiences for sleep-dependent memory processing. While our results do not imply that consolidation processes are unique to sleep, they do suggest that there may be a unique memory function of sleep under certain conditions. Salience, novelty, and future relevance are all factors that influence overnight memory processing in animals and humans [6,15,16,30,31], and are thus likely involved in shaping which experiences get tagged for consolidation during sleep. We intentionally used non-affective stimuli in the current experiment so that we could isolate generalised neurocognitive markers of mnemonic tagging. However, individual differences in the perceived novelty or salience of word-object pairings may have influenced which memories were tagged for consolidation during sleep. Delineating the impact of such variables on our ability to decode neural tagging operations at learning is clearly a priority area for future research.”* – Pages 9-10

To the Reviewer’s question of how tagging effects would play out over longer delays, this is a critical question that will need to be addressed in future research. Behaviourally, a number of studies have shown stronger sleep effects following a 24-hour sleep-first delay vs a 24-hour wake-first delay for emotionally neutral stimuli such as word pairs or spatial memory for the location of neutral images (e.g., Gais et al., 2006; Talamini et al., 2008; Payne et al., 2012; Denis et al., 2020). This could imply that tags set in the wake-first group decayed over the wake interval and so were not acted on by sleep. Given the uninteresting nature of the stimuli, any tagging signal was likely to be weak. We would hypothesise that should a sufficiently strong tag be set, this would persist across wakefulness until a sleep period where the tag could be acted on to consolidate the event. Indeed, it is possible in the current study that individual differences in stimuli that participants considered interesting or meaningful could have influenced which items were tagged for consolidation across sleep. While we can’t know this for sure, future studies could experimentally manipulate this (e.g., using affective vs non-affective stimuli). We have expanded on these ideas in the discussion as follows:

*“In the current study, sleep occurred soon after learning, making it unclear whether tags established at learning would endure over longer delays. Past research has found that sleep-memory effects are reduced over a 24-hour wake-first delay compared to a 24-hour sleep-first delay, which could be interpreted as learning-related tags fading over the initial wake interval [36,48–50]. These studies have typically used emotionally neutral stimuli such as word-pairs or object locations, meaning the initial tag would likely have been relatively weak. We hypothesise that a sufficiently strong tag at learning (e.g., corresponding to a highly salient or stressful experience) would elicit a strong enough tag for it to endure across a longer delay, permitting targeted consolidation when sleep is eventually reached.”* – Pages 11

# Comment 2

Can the authors examine the SO-SP phase alignment with the theta at encoding? it might be interesting to see if the remembered vs forgotten theta ratio (or some such metric) might relate to any phase alignment metric between SOs and SPs. This would speak to the literature on 'optimal' phase coupling between SOs and SPs.

# Response

Thank you for this interesting suggestion, however we found no association between encoding-related theta power (remembered > forgotten) and coupling phase (no clusters formed).

# Comment 3

There is a growing literature on the role of rem sleep for forgetting, synaptic pruning, rem theta and alpha bursts, the refine and rescue hypothesis (shuster 2025). What is the role of REM sleep and in particular rem theta in these data? Speculation is fine.

# Response

Our focus on NREM sleep was guided by active systems consolidation theory, which primarily implicates NREM sleep as a facilitator of memory consolidation. Additionally, by using a daytime nap, a thorough investigation of REM sleep wasn’t possible given low or even no REM sleep being common in daytime naps. We agree with the Reviewer however that there is growing interest in the possible role of REM sleep, especially given suggestions that REM sleep is involved in the selective consolidation of emotional memories (e.g., Kim & Payne, 2020). We have speculated on this in the discussion as follows:

*“We focused on SO-spindle coupling during non-rapid eye movement sleep as a putative marker of sleep-dependent consolidation, based on long-standing evidence that these oscillations are involved in memory reactivation and systems-level consolidation [3,25]. However, there is evidence of mnemonic processing during rapid eye movement (REM) sleep, with some research suggesting that REM sleep supports the selective consolidation of emotionally salient memories [45–47]. A thorough examination of REM sleep in this dataset was not possible, owing to short REM sleep durations common to daytime napping paradigms. Follow-up research using an overnight design to sufficiently quantify REM sleep is therefore needed to understand how memory tagging operations interact with memory reprocessing during REM.”* – Page 11

# Comment 4

Theta gamma coupling during encoding has been frequently reported, can the authors examine the encoding signal more to characterize the encoding tag?

# Response

We agree with the Reviewer that an analysis of theta-gamma coupling would be interesting, however we determined that such an analysis was not feasible in this study. Gamma-band oscillations are very low amplitude, and have a poor SNR as measured by EEG. As such, this question would be better suited to a different methodology such as MEG.
