# Supplementary material for: Theta oscillations tag episodic memories for sleep-dependent consolidation
Source: PLoS Biol. 2026 Aug 27;24(8):e3003938. doi: 10.1371/journal.pbio.3003938 (PMC13521381; doi:10.1371/journal.pbio.3003938)
Supplement: Supplementary file 8 [file pbio.3003938.s015.docx]

# Reviewer 2

The authors have sufficiently addressed my previous concerns regarding the conceptual novelty (theta, sleep, memory) and robustness of the analyses (EEG slope, decoding, coupled spindle density). I have three remaining question:

## Comment 1

First, there remains a critical gap between the neural decoding and theta tagging mechanism. The authors first showed that EEG-based decoding can distinguish between memories that are remembered after sleep vs. wake, followed by examining theta power. I would wonder whether theta power may have driven the MVPA sleep vs. wake decoding? The authors used a correlational approach to link theta and MVPA, but it would be more convincing and more straightforward to examine whether theta contributed to MVPA.

## Response

We thank for the reviewer for the suggestion to carry out our MVPA analyses on the theta power time-frequency representation (TFR). Although we considered this, we ultimately decided against this approach on the basis that it would work against the primary strengths of the MVPA method. By applying MVPA to the raw EEG timeseries, it is able to take advantage of the full multivariate spatiotemporal signal. The theta TFR is a narrowband filtered signal (3-8Hz) that has also been temporally smoothed as a function of the time-frequency decomposition. If we were to run MVPA on the theta TFR, potentially important information contributing to classification may have been removed.

Therefore, we believe our current approach to be the most optimal. By performing MVPA on the raw EEG timeseries, we are providing the classifier with the most information possible in order to decode tagging operations. The TFR complements this by directly testing the involvement of a theoretically relevant neural oscillation. This approach, which capitalises on the strengths of both approaches, is widely used in the literature (e.g., Tarrasó et al (2026); Schreiner et al (2021); Weisz et al (2020); Kerrén et al (2018) for just a few recent examples).

Our correlational approach also revealed an especially interesting time-lagged correlation between theta power and MVPA accuracy that would not have been revealed had MVPA been applied directly to the theta TFR. We showed that early increases in theta power predicted *later* classification accuracy. This is an important observation which we speculate may reveal a specific role for theta in signalling initial event salience, which then triggers a downstream tag to be set. We raise this possibility in the discussion as follows:

*“Interestingly, evidence of tagging for sleep-dependent consolidation emerged in our classification analyses with a latency of ~1 s. This is temporally aligned with classifiers that have tracked the time course of memory reinstatement during retrieval in humans [39,40], and therefore suggests that neural tags for sleep-specific memory processing may emerge from early wake replay events. This notion is aligned with recent work in rodent models showing that awake replay shortly after a learning event itself constitutes a tagging mechanism for memory processing during later sleep [41].* ***In the context of the current study, early increases in theta activity and associated binding processes during episodic learning may prompt rapid replay events that constitute a tag for targeted overnight memory processing.”*** – Page 10

## Comment 2

Why there is significant decoding between visit 1 and visit 2? This is the learning order and should be comparable, right?

## Response

The ability for the classifier to successfully discriminate between experimental visit is most likely explained by subtle between-visit differences in EEG preparation, such as small differences in impedance level or EEG electrode position. Because these small differences will ultimately impact the EEG signal, we were not surprised that a classifier picked up on this and was able to distinguish between experimental visits. However, because we counterbalanced sleep/wake order, we were able to dissociate generic between visit differences from real differences in how memories were tagged prior to either a sleep or wake delay via our subtraction approach. This is described in the methods section of the manuscript as follows:

*“An important caveat of our approach is that, by necessity, the sleep and wake conditions were performed on separate experimental days. Non-specific between-session differences, such as differences in impedance levels and the exact positioning of electrodes could thus be picked up by the classifier. It was therefore crucial to ensure that any signal discriminating between the sleep and wake conditions did not more parsimoniously reflect significant classification of the experimental sessions. To account for this, a set of control analyses were carried out. First, we ran the classifier again, but this time distinguishing trials that were forgotten after sleep compared to forgotten after wake (i.e., the Forgotten classifier). By comparing this classifier to the first Remember classifier, we could look for periods where classification accuracy was significantly higher for later remembered vs forgotten trials. If classification accuracy was higher for one vs the other, this would speak against more generic between visit differences, because we would not expect broad between-sessions differences to impact the memory conditions differently. As a second control analysis, we ran a third classifier to discriminate between Visit 1 and Visit 2 (the Visit classifier). This is dissociable from the sleep and wake condition because the assignment of visit order (sleep first or wake first) was counterbalanced across participants.”* – Page 17

## Comment 3

Inspecting figure 2 B suggests that theta power did not differ between remember vs. forgotten conditions. Does it contradict with previous findings showing that theta predicts SME during wakefulness? If so, how to reconcile?

## Response

Figure 2B shows the theta power time series after subtracting forgotten trials from remembered trials, meaning results reflect memory specific fluctuations in theta oscillatory power. As reported, theta power was significantly greater than zero in the sleep condition:

*“The increase in theta power was significantly greater than zero in the sleep condition (5.68% ± 8.09%; t (30) = 3.90, p < .001, d = 0.70), but not in the wake condition (-2.73% ± 12.18%; t (30) = -1.25, p = .22, d = -0.22).”* – Page 7

Further analysis confirms that this does indeed reflect significantly higher theta power for subsequently remembered trials (13.66%, ± 15.72%) compared to subsequently forgotten (7.98% ± 14.14%); *t* (30) = 3.90, *p* < .001) trials in the sleep condition. However the reviewer is correct that there was no significant difference between remembered and forgotten trials in the wake condition (*t* (30) = -1.25, *p* = .22).

In terms of reconciling these findings with the wider literature, it is important to note that these results are examining subsequent memory after a 2-hour delay. This differs from most previous studies who examine memory immediately following learning. Therefore, our findings are most consistent with the idea that when learning is followed by a consolidation period, learning-related theta power reflects a tagging of memories for subsequent consolidation during sleep specifically.
