# Supplementary material for: Theta oscillations tag episodic memories for sleep-dependent consolidation
Source: PLoS Biol. 2026 Aug 27;24(8):e3003938. doi: 10.1371/journal.pbio.3003938 (PMC13521381; doi:10.1371/journal.pbio.3003938)
Supplement: Peer review history

# Editor report: Decision Letter 0

**DOI:** 10.1371/journal.pbio.3003938.r001

**Authors:** Lucas Smith

19 Dec 2025

Dear Dr Denis,

Thank you for submitting your manuscript entitled "Theta oscillations tag memories for sleep-dependent consolidation" for consideration as a Research Article by PLOS Biology.

Your manuscript has now been evaluated by the PLOS Biology editorial staff as well as by an academic editor with relevant expertise and I am writing to let you know that we would like to send your submission out for external peer review.

However, before we can send your manuscript to reviewers, we need you to complete your submission by providing the metadata that is required for full assessment. To this end, please login to Editorial Manager where you will find the paper in the 'Submissions Needing Revisions' folder on your homepage. Please click 'Revise Submission' from the Action Links and complete all additional questions in the submission questionnaire.

Once your full submission is complete, your paper will undergo a series of checks in preparation for peer review. After your manuscript has passed the checks it will be sent out for review. To provide the metadata for your submission, please Login to Editorial Manager (https://www.editorialmanager.com/pbiology) within two working days, i.e. by Dec 23 2025 11:59PM.

If your manuscript has been previously peer-reviewed at another journal, PLOS Biology is willing to work with those reviews in order to avoid re-starting the process. Submission of the previous reviews is entirely optional and our ability to use them effectively will depend on the willingness of the previous journal to confirm the content of the reports and share the reviewer identities. Please note that we reserve the right to invite additional reviewers if we consider that additional/independent reviewers are needed, although we aim to avoid this as far as possible. In our experience, working with previous reviews does save time.

If you would like us to consider previous reviewer reports, please edit your cover letter to let us know and include the name of the journal where the work was previously considered and the manuscript ID it was given. In addition, please upload a response to the reviews as a 'Prior Peer Review' file type, which should include the reports in full and a point-by-point reply detailing how you have or plan to address the reviewers' concerns.

During the process of completing your manuscript submission, you will be invited to opt-in to posting your pre-review manuscript as a bioRxiv preprint. Visit http://journals.plos.org/plosbiology/s/preprints for full details. If you consent to posting your current manuscript as a preprint, please upload a single Preprint PDF.

Feel free to email us at plosbiology@plos.org if you have any queries relating to your submission.

**As a last note - the PLOS Biology editorial office will be closed from December 22 to January 2nd, and so we will need to wait until early in the new year to begin inviting reviewers for your paper. Our operations team will still be working, and will complete the necessary metadata checks while I am away.

Kind regards,

Luke

Lucas Smith, Ph.D.

Senior Editor

PLOS Biology

lsmith@plos.org

# Peer review: Decision Letter 1

**DOI:** 10.1371/journal.pbio.3003938.r002

**Authors:** Lucas Smith

12 Feb 2026

Dear Dr Denis,

Thank you for your patience while your manuscript "Theta oscillations tag memories for sleep-dependent consolidation" was peer-reviewed at PLOS Biology. It has now been evaluated by the PLOS Biology editors, an Academic Editor with relevant expertise, and by several independent reviewers.

As you will see below, the reviewers report that the findings reported here are potentially interesting, but they also raise a number of important concerns that we think will need to be thoroughly addressed before we can consider your study for publication at PLOS Biology. While we think that all the reviewer comments should be addressed, we would like to emphasize that we think that it will be essential for you to provide the additional analyses proposed by reviewer 1 (as well as providing additional justifications and clarifications as requested), which we think this is needed to support the conclusions of this study and to increase the impact of this work.

Given the extent of revision needed, we cannot make a decision about publication until we have seen the revised manuscript and your response to the reviewers' comments. Your revised manuscript is likely to be sent for further evaluation by all or a subset of the reviewers.

In addition to these revisions, you will need to complete some formatting changes, which you will receive in a follow up email. A member of our team will be in touch with a set of requests shortly.

We expect to receive your revised manuscript within 3 months. Please email us (plosbiology@plos.org) if you have any questions or concerns, or would like to request an extension.

At this stage, your manuscript remains formally under active consideration at our journal; please notify us by email if you do not intend to submit a revision so that we may withdraw it.

**IMPORTANT - SUBMITTING YOUR REVISION**

Your revisions should address the specific points made by each reviewer. Please submit the following files along with your revised manuscript:

1. A 'Response to Reviewers' file - this should detail your responses to the editorial requests, present a point-by-point response to all of the reviewers' comments, and indicate the changes made to the manuscript.

*NOTE: In your point-by-point response to the reviewers, please provide the full context of each review. Do not selectively quote paragraphs or sentences to reply to. The entire set of reviewer comments should be present in full and each specific point should be responded to individually, point by point.

You should also cite any additional relevant literature that has been published since the original submission and mention any additional citations in your response.

2. In addition to a clean copy of the manuscript, please also upload a 'track-changes' version of your manuscript that specifies the edits made. This should be uploaded as a "Revised Article with Changes Highlighted" file type.

*Re-submission Checklist*

When you are ready to resubmit your revised manuscript, please refer to this re-submission checklist: https://plos.io/Biology_Checklist

To submit a revised version of your manuscript, please go to https://www.editorialmanager.com/pbiology/ and log in as an Author. Click the link labelled 'Submissions Needing Revision' where you will find your submission record.

Please make sure to read the following important policies and guidelines while preparing your revision:

*Published Peer Review*

Please note while forming your response, if your article is accepted, you may have the opportunity to make the peer review history publicly available. The record will include editor decision letters (with reviews) and your responses to reviewer comments. If eligible, we will contact you to opt in or out. Please see here for more details:

https://blogs.plos.org/plos/2019/05/plos-journals-now-open-for-published-peer-review/

*PLOS Data Policy*

Please note that as a condition of publication PLOS' data policy (http://journals.plos.org/plosbiology/s/data-availability) requires that you make available all data used to draw the conclusions arrived at in your manuscript. If you have not already done so, you must include any data used in your manuscript either in appropriate repositories, within the body of the manuscript, or as supporting information (N.B. this includes any numerical values that were used to generate graphs, histograms etc.). For an example see here: http://www.plosbiology.org/article/info%3Adoi%2F10.1371%2Fjournal.pbio.1001908#s5

*Blot and Gel Data Policy*

We require the original, uncropped and minimally adjusted images supporting all blot and gel results reported in an article's figures or Supporting Information files. We will require these files before a manuscript can be accepted so please prepare them now, if you have not already uploaded them. Please carefully read our guidelines for how to prepare and upload this data: https://journals.plos.org/plosbiology/s/figures#loc-blot-and-gel-reporting-requirements

*Protocols deposition*

To enhance the reproducibility of your results, we recommend that if applicable you deposit your laboratory protocols in protocols.io, where a protocol can be assigned its own identifier (DOI) such that it can be cited independently in the future. Additionally, PLOS ONE offers an option for publishing peer-reviewed Lab Protocol articles, which describe protocols hosted on protocols.io. Read more information on sharing protocols at https://plos.org/protocols?utm_medium=editorial-email&utm_source=authorletters&utm_campaign=protocols

Thank you again for your submission to our journal. We hope that our editorial process has been constructive thus far, and we welcome your feedback at any time. Please don't hesitate to contact us if you have any questions or comments.

Sincerely,

Luke

Lucas Smith, Ph.D.

Senior Editor

PLOS Biology

lsmith@plos.org

------------------------------------

REVIEWS:

Reviewer #1: Summary

This study aimed to identify neurophysiological signatures of memory "tagging" during learning that predict selective memory reprocessing during subsequent sleep. EEG-derived theta-band activity during encoding (3-8 Hz) differentiates items that will later be remembered after sleep versus wake. The magnitude of this theta response during learning predicts the density of slow-oscillation-coupled sleep spindles during the post-learning nap, a commonly used proxy for sleep-dependent plasticity, over parieto-occipital regions. SO-spindle coupling density over central regions is associated with better post-sleep memory performance.

Overall evaluation

Overall, the manuscript is clearly written, focused, and communicates a strong central message. The experimental design is straightforward although some of the analytical decisions are in need of further justification. That said, the paper would benefit from additional integrative analyses to strengthen their interpretive claims (e.g., explicitly testing the proposed theta → coupling → behavior pathway). In addition, several important limitations of the paradigm and of inference from scalp EEG (especially regarding topographic differences and claims about tagging specificity) should be acknowledged more explicitly in the Discussion.

MAJOR COMMENTS

1.In Figure 1D, the authors use three classifiers: remembered (sleep vs. wake), forgotten (sleep vs. wake), and visit (1 vs. 2). They are combined via a double-subtraction logic to isolate a tagging-related signal. While conservative, it is not obvious that this approach provides the most direct test of the tagging hypothesis as formulated in the Introduction.

If memories are "tagged" during encoding such that they are preferentially consolidated over others during sleep, in my opinion a more straightforward contrast remembered vs. forgotten items within each consolidation condition. Classifiers trained separately on remembered vs. forgotten trials for sleep and wake would allow a direct assessment of whether encoding-related signals are selectively enhanced for memories benefiting from sleep (i.e., sleep > wake). Under this logic, a separate visit classifier would not be strictly necessary if condition order was balanced across participants. The authors state in the respective methods section that their goal is to "distinguish memories retained over sleep from memories retained over wakefulness" (p. 7) which, in my opinion, is better expressed in a sleep > wake. This logic also applies to the theta power differences in panel Fig. 2B. Thus, please explain and justify your choice.

2.Related to the previous comment, to better understand the processes underlying state-specific ex ante tagging, it would also be important to know when participants were told whether they would be napping or not, and whether they were told (or expected) sleep/wake conditions to be counterbalanced.

3.A central claim of the manuscript is that theta-band activity during encoding serves as a memory tag that predicts subsequent sleep-dependent reprocessing, which in turn supports memory retention. However, this proposed pathway is currently supported only by two separate correlations: theta power with SO-spindle coupling (Fig. 2D) and SO-spindle coupling with retention (Fig. 2E).

Given the explicit directional logic of the model, a mediation analysis testing whether SO-spindle coupling mediates the relationship between encoding-related theta power and memory performance would substantially strengthen the conclusions. At minimum, it should be reported whether encoding-related theta power directly correlates with post-sleep retention, and how this relationship changes when spindle coupling is included as a predictor. If such analyses were not performed, the causal framing should be toned down and included as a limitation in the discussion.

4.To support the claim that theta-band activity reflects a sleep-specific tagging mechanism rather than general encoding strength, additional control analyses are warranted. Specifically, it would be informative to test whether encoding-related theta power also differentiates remembered vs. forgotten items at immediate retrieval, and whether this theta signal predicts SO-spindle coupling in the same way. Repeating analyses analogous to Fig. 2B and Fig. 2D using immediate memory outcomes would help establish that the observed theta-spindle relationship is selective to sleep-dependent consolidation rather than reflecting a general marker of successful encoding.

MINOR COMMENTS

1.Please specify the exact software and functions used for the statistical analyses, for instance for the (multivariate) robust linear regressions, to improve reproducibility.

2.Spindles occurring near the SO up-state (or down-to-up transition) are often considered most relevant for memory consolidation. Did the authors repeat the analyses using only up-state-locked spindles, and if so, were the effects comparable or stronger? If not, please justify the choice to include all spindles between SO zero crossings.

3.Analysis of immediate vs. delayed performance (Fig. 2C): Rather than arguing for the absence of group differences at immediate recall, a more appropriate approach would be to include Session (immediate vs. delayed) and Condition (sleep vs. wake) in a single model (e.g., two-way ANOVA or linear mixed-effects model) and explicitly test their interaction. Please clarify whether the interaction would be significant in this case.

4.The analysis for theta power was hypothesis driven. As a control, the authors also performed the classifications for the broadband spectra. Was the theta effect also present for this broadband analysis?

5.For non-significant correlations involving other spindle, SO, or coupling parameters, described in the last paragraphs of the results section, please report p-values (and effect sizes where appropriate), either in the main text or supplementary materials, particularly when interpreting null findings.

6.Please provide a supplementary table summarizing spindle and SO characteristics (e.g., density, amplitude, frequency, duration) to support the validity of the detection procedures.

7.The Discussion should explicitly address why SO-spindle coupling correlates with encoding-related theta over occipital regions but with behavioral retention over central regions. Given frequent claims that replay is local, this spatial dissociation requires cautious interpretation.

8.Replay processes also occur during wake. Please discuss how the apparent selectivity of encoding-related theta for sleep-dependent consolidation should be interpreted in this broader context.

9.The classifier distinguishes conditions only ~1 s after word onset and for a brief time window (~150 ms; Fig. 2A). Please discuss why tagging-related information emerges at this latency and why it is so temporally restricted.

10.What was the range of trials per condition that could be used for analysis? (p. 17)

11.The reasons provided for why the results do not reflect individual differences are not very convincing. It might be more interesting to test whether performance in the preliminary session was related to (coupled) spindle density.

12.The focus on correctly recalled, elaborate associations should be made more explicit in the title and the abstract.

13.What was the range of channels interpolated across participants? Given that only 14 electrodes were used, interpolation may have exaggerated the reliability of the signals.

14.Did the theta effect also emerge from the broadband analysis that was used to explore correlations with other frequency bands?

Reviewer #2: Thank you for the opportunity to review this manuscript on the role of scalp EEG theta activity in sleep-dependent memory consolidation. I read this work with great interest. The authors reported a single experiment with a cross-over, within-participants design, in which participants completed encoding and retrieval tests separated by a daytime nap or a period of wakefulness. Employing a variant of the subsequent memory effect approach and multivariate decoding (remember_sleep vs. wake decoding, forgotten_sleep vs.wake decoding, visit 1 vs. visit 2 decoding), results showed that theta power (but not other frequency bands) during encoding predicted memory performance in the sleep condition. Moreover, and perhaps most interestingly, theta power during encoding predicted subsequent slow oscillation - spindle coupling index during sleep, and then predicted sleep-dependent memory consolidation.

I have some comments about the rationale, analyses, and interpretation of the results.

The role of theta power in episodic memories has received much attention in human memory research. It is almost well-established that theta power could predict various types of memory, at least during wakefulness (Herweg et al., 2020, Trends in Cognitive Sciences). This brings up the first question: What is the theoretical contribution of the current study? Although the study argues for the "memory tagging" mechanism, what the results showed were essentially the same as in previous research: enhanced theta power was associated with successful memory. Indeed, the study is probably the first demonstrating that theta activity predicted sleep-dependent memory consolidation, but then the results did not replicate the well-established findings that theta power could predict memory in wakefulness. Thus, the theoretical contributions remain unclear, and the significant discrepancy remains unresolved.

Conceptually, the manuscript argues that theta activity is a memory tagging mechanism. This is indeed an important question that still awaits more research. However, given that the behavioral and decoding analyses were performed at a condition level (remember vs. forgotten) instead of an item level, the results are not much different from previous findings showing that EEG/ERP activities during encoding were associated with subsequent retrieval (see work from Hanslmayr, Paller, Mecklinger on the EEG/ERP correlates of subsequent memory effects). The manuscript needs more elaboration on the novelty and how much new information the data provides.

The value and implication of the multivariate decoding approach need to be further elaborated. Given that the decoding analyses were all conducted at a condition level instead of an item/memory content level, how the decoding analyses can be informative beyond traditional condition/group-based EEG/ERP analyses remains unclear. Furthermore, given that there were no independent memory/functional localizer tasks (see Schreiner et al., 2021, Nature Communications) that can speak to memory content, how to best understand the decoding results and the theta activity remains unclear.

There are concerns regarding the precision of terms and quantification of the key neural activity, theta activities. The manuscript used theta power, theta oscillation almost interchangeably. However, recent methods can effectively quantify the theta oscillation from the background EEG spectral slope. To provide new theoretical insights, at least the analyses shall try to dissociate oscillatory activity from the background slope or the "tilt" (again, see Herweg et al., 2020, Trends in Cognitive Sciences), to better qualify the strong argument on the role of theta in so-called "memory tagging" for subsequent sleep-dependent memory consolidation.

One novel result is that the theta activity during wakeful encoding was related to SO-spindle coupling during sleep. The current study calculated SO-spindle coupling density. However, a recent meta-analysis suggests that coupling percentage was a poor predictor of memory consolidation. Instead, the coupling strength and coupling phases are stronger and more reliable predictors (Ng et al., 2025, elife). The results and argument will be more convincing if theta activity can also predict coupling strength/phase, and then predict memory consolidation.

Reviewer #3: Denis and colleagues examined whether learning paired associates imparted a specific tag that was related to sleep consolidation vs wake consolidation and how these tags might be associated with sleep oscillations known to be related to memory and subsequent retrieval. The study is well done and the paper is well written. Results show significant distinctions between remembered items across wake and sleep but not across immediate test, which is a critical control. I believe these findings will open new avenues of exploration between waking brain activity and subsequent sleep consolidation. I have some questions about the interpretation and some requests for further analyses.

1.Although the results are quite clear, there is a little confusion in the writing about the results that can hopefully be cleared up. Such as this sentence: these findings are consistent with the idea that memories are tagged at learning for selective consolidation during later sleep.

When I read that, it sounds like the brain is tagging material that it already knows will be consolidated by sleep and not wake. I assume this is not what the authors intend. Can you find a way to talk about this with less of a mindreading implication? For instance, the learning material is tagged at encoding irrespective of what consolidation process follows, and that the retrieval state of the memory tag is transformed by the circumstances of consolidation: sleep vs wake.

Now reading the discussion I see that you do in fact mean that the brain triages memories for sleep and not wake… hmm… the results show some benefits of sleep over wake for retention, but they are not massive benefits. And this was a simple pair associates task without a lot of complicated relations to remember. Therefore, the idea that some information should be stored away for a time when sleep can consolidate while most of the memories can be tagged and processed during either wake or sleep, seems like a stretch in logic to me. If a 24 hour protocol was used with a sleep first (trained at night) vs wake first (trained in am) comparison, would the authors predict that the theta-save-it-for-sleep effect would be eliminated because both groups eventually slept? Or would the authors say that the tag would decay?

2.Can the authors examine the SO-SP phase alignment with the theta at encoding? it might be interesting to see if the remembered vs forgotten theta ratio (or some such metric) might relate to any phase alignment metric between SOs and SPs. This would speak to the literature on 'optimal' phase coupling between SOs and SPs.

3.There is a growing literature on the role of rem sleep for forgetting, synaptic pruning, rem theta and alpha bursts, the refine and rescue hypothesis (shuster 2025). What is the role of REM sleep and in particular rem theta in these data? Speculation is fine.

4.Theta gamma coupling during encoding has been frequently reported, can the authors examine the encoding signal more to characterize the encoding tag?

# Author comment: Author response to Decision Letter 2

**DOI:** 10.1371/journal.pbio.3003938.r003

27 May 2026

Attachment Submitted filename: SUBMITTED_Response to reviews.docx

# Peer review: Decision Letter 2

**DOI:** 10.1371/journal.pbio.3003938.r004

**Authors:** Lucas Smith

9 Jul 2026

Dear Dr Denis,

Thank you for your patience while we considered your revised manuscript "Theta oscillations tag episodic memories for sleep-dependent consolidation" for consideration as a Research Article at PLOS Biology. Your revised study has now been evaluated by the PLOS Biology editors, the Academic Editor and by two of the original reviewers.

In light of the reviews, which you will find at the end of this email, we are pleased to offer you the opportunity to address the remaining points from reviewer 2 in a revision that we anticipate should not take you very long. We will then assess your revised manuscript and your response to the reviewers' comments with our Academic Editor aiming to avoid further rounds of peer-review, although we might need to consult with the reviewers, depending on the nature of the revisions.

In addition to these revisions, you may need to complete some formatting changes, which you will receive in a follow up email. A member of our team will be in touch with a set of requests shortly. If you do not receive a separate email within a few days, please assume that checks have been completed, and no additional changes are required.

We expect to receive your revised manuscript within 1 month. Please email us (plosbiology@plos.org) if you have any questions or concerns, or would like to request an extension.

At this stage, your manuscript remains formally under active consideration at our journal; please notify us by email if you do not intend to submit a revision so that we withdraw the manuscript.

**IMPORTANT - SUBMITTING YOUR REVISION**

Your revisions should address the specific points made by each reviewer. Please submit the following files along with your revised manuscript:

1. A 'Response to Reviewers' file - this should detail your responses to the editorial requests, present a point-by-point response to all of the reviewers' comments, and indicate the changes made to the manuscript.

*NOTE: In your point-by-point response to the reviewers, please provide the full context of each review. Do not selectively quote paragraphs or sentences to reply to. The entire set of reviewer comments should be present in full and each specific point should be responded to individually.

You should also cite any additional relevant literature that has been published since the original submission and mention any additional citations in your response.

2. In addition to a clean copy of the manuscript, please also upload a 'track-changes' version of your manuscript that specifies the edits made. This should be uploaded as a "Revised Article with Changes Highlighted " file type.

*Resubmission Checklist*

When you are ready to resubmit your revised manuscript, please refer to this resubmission checklist: https://plos.io/Biology_Checklist

To submit a revised version of your manuscript, please go to https://www.editorialmanager.com/pbiology/ and log in as an Author. Click the link labelled 'Submissions Needing Revision' where you will find your submission record.

Please make sure to read the following important policies and guidelines while preparing your revision:

*Published Peer Review*

Please note while forming your response, if your article is accepted, you may have the opportunity to make the peer review history publicly available. The record will include editor decision letters (with reviews) and your responses to reviewer comments. If eligible, we will contact you to opt in or out. Please see here for more details:

https://blogs.plos.org/plos/2019/05/plos-journals-now-open-for-published-peer-review/

*PLOS Data Policy*

Please note that as a condition of publication PLOS' data policy (http://journals.plos.org/plosbiology/s/data-availability) requires that you make available all data used to draw the conclusions arrived at in your manuscript. If you have not already done so, you must include any data used in your manuscript either in appropriate repositories, within the body of the manuscript, or as supporting information (N.B. this includes any numerical values that were used to generate graphs, histograms etc.). For an example see here: http://www.plosbiology.org/article/info%3Adoi%2F10.1371%2Fjournal.pbio.1001908#s5

*Blot and Gel Data Policy*

We require the original, uncropped and minimally adjusted images supporting all blot and gel results reported in an article's figures or Supporting Information files. We will require these files before a manuscript can be accepted so please prepare them now, if you have not already uploaded them. Please carefully read our guidelines for how to prepare and upload this data: https://journals.plos.org/plosbiology/s/figures#loc-blot-and-gel-reporting-requirements

*Protocols deposition*

To enhance the reproducibility of your results, we recommend that if applicable you deposit your laboratory protocols in protocols.io, where a protocol can be assigned its own identifier (DOI) such that it can be cited independently in the future. Additionally, PLOS ONE offers an option for publishing peer-reviewed Lab Protocol articles, which describe protocols hosted on protocols.io. Read more information on sharing protocols at https://plos.org/protocols?utm_medium=editorial-email&utm_source=authorletters&utm_campaign=protocols

Thank you again for your submission to our journal. We hope that our editorial process has been constructive thus far, and we welcome your feedback at any time. Please don't hesitate to contact us if you have any questions or comments.

Sincerely,

Luke

Lucas Smith, Ph.D.

Senior Editor

PLOS Biology

lsmith@plos.org

----------------------------------------------------------------

REVIEWS:

Reviewer #1: The authors have satisfactorily revised the ms which, in my view, is now acceptable for publication.

Reviewer #2: The authors have sufficiently addressed my previous concerns regarding the conceptual novelty (theta, sleep, memory) and robustness of the analyses (EEG slope, decoding, coupled spindle density).

I have three remaining question: First, there remains a critical gap between the neural decoding and theta tagging mechanism. The authors first showed that EEG-based decoding can distinguish between memories that are remembered after sleep vs. wake, followed by examining theta power. I would wonder whether theta power may have driven the MVPA sleep vs. wake decoding? The authors used a correlational approach to link theta and MVPA, but it would be more convincing and more straightforward to examine whether theta contributed to MVPA.

A second question is related to the decoding analyses: why there is significant decoding between visit 1 and visit 2? This is the learning order and should be comparable, right?

Third, inspecting figure 2 B suggests that theta power did not differ between remember vs. forgotten conditions. Does it contradict with previous findings showing that theta predicts SME during wakefulness? If so, how to reconcile?

# Author comment: Author response to Decision Letter 3

**DOI:** 10.1371/journal.pbio.3003938.r005

13 Jul 2026

Attachment Submitted filename: SUBMITTED_Revision2_Response to reviews.docx

# Editor report: Decision Letter 3

**DOI:** 10.1371/journal.pbio.3003938.r006

**Authors:** Lucas Smith

24 Jul 2026

Dear Dr Denis,

Thank you for the submission of your revised Research Article "Theta oscillations tag episodic memories for sleep-dependent consolidation" for publication in PLOS Biology. On behalf of my colleagues and the Academic Editor, Simon Hanslmayr, I am pleased to say that we are satisfied by this most recent revision and we can in principle accept your manuscript for publication, provided you address any remaining formatting and reporting issues. These will be detailed in an email you should receive within 2-3 business days from our colleagues in the journal operations team; no action is required from you until then. Please note that we will not be able to formally accept your manuscript and schedule it for publication until you have completed any requested changes.

**IMPORTANT - As you address any production related requests, to come, I also ask that you attend to the following editorial requests:

1) ETHICS STATEMENT: Please update the ethics statement in your materials and methods section to indicate whether this study was conducted in accordance with the principles expressed in the Declaration of Helsinki.

2) DATA: Thank you for providing the underlying data and code for your study as a OSF deposition. Please add a sentence to each figure legends, pointing readers to this underlying data. For example, you can add the sentence 'The data underlying this figure are available at https://doi.org/10.17605/OSF.IO/TPWVB)

Please take a minute to log into Editorial Manager at http://www.editorialmanager.com/pbiology/, click the "Update My Information" link at the top of the page, and update your user information to ensure an efficient production process.

PRESS

We frequently collaborate with press offices. If your institution or institutions have a press office, please notify them about your upcoming paper at this point, to enable them to help maximise its impact. If the press office is planning to promote your findings, we would be grateful if they could coordinate with biologypress@plos.org. If you have previously opted in to the early version process, we ask that you notify us immediately of any press plans so that we may opt out on your behalf.

We also ask that you take this opportunity to read our Embargo Policy regarding the discussion, promotion and media coverage of work that is yet to be published by PLOS. As your manuscript is not yet published, it is bound by the conditions of our Embargo Policy. Please be aware that this policy is in place both to ensure that any press coverage of your article is fully substantiated and to provide a direct link between such coverage and the published work. For full details of our Embargo Policy, please visit http://www.plos.org/about/media-inquiries/embargo-policy/.

Thank you again for choosing PLOS Biology for publication and supporting Open Access publishing. We look forward to publishing your study.

Sincerely,

Luke

Lucas Smith, Ph.D.

Senior Editor

PLOS Biology

lsmith@plos.org
